# Social–ecological transformations towards sustainability in drylands: A conceptual framework with examples from the Karamoja cluster in East Africa

**DOI:** 10.1007/s13280-026-02389-9

**Published:** 2026-04-24

**Authors:** Aida Bargués-Tobella, Per Knutsson, Göran Bostedt, Agneta Hörnell, Kristina Lindvall, Denis Mpairwe, Stephen Mureithi, Gert Nyberg, Ylva Nyberg, Philemon Ong’ao Ng’asike, Barbara Schumann, Alice Turinawe, Tor-Gunnar Vågen, Leigh Ann Winowiecki, Ewa Wredle, Ingrid Öborn

**Affiliations:** 1 Agrotecnio-CERCA Center, Av. Rovira Roure 191, 25198 Lleida, Spain; 2 Department of Forest Ecology and Management, Swedish University of Agricultural Sciences (SLU), 901 83 Umeå, Sweden; 3 School of Global Studies, University of Gothenburg, 405 30 Göteborg, Sweden; 4 Centre for Environmental and Resource Economics, CERE, Umeå School of Business, Economics and Statistics (USBE), Umeå University, 901 87 Umeå, Sweden; 5 Department of Forest Economics, Swedish University of Agricultural Sciences (SLU), Umeå, Sweden; 6 Department of Food, Nutrition and Culinary Science, Umeå University, 901 87 Umeå, Sweden; 7 Department of Epidemiology and Global Health, Umeå University, 901 87 Umeå, Sweden; 8 Department of Agricultural Production, Makerere University, P.O. Box 7062, Kampala, Uganda; 9 Department of Land Resource Management and Agricultural Technology, University of Nairobi, P.O. Box 29053-00625, Nairobi, Kenya; 10 Department of Crop Production Ecology, Swedish University of Agricultural Sciences (SLU), 750 07 Uppsala, Sweden; 11 Department of Health and Caring Sciences, Linnaeus University, 391 82 Kalmar, Sweden; 12 Department of Agribusiness and Natural Resource Economics, College of Agricultural and Environmental Sciences, Makerere University, P.O. Box 7062, Kampala, Uganda; 13 World Agroforestry (CIFOR-ICRAF), P.O. Box 30677, Nairobi, 00100 Kenya; 14 Department of Applied Animal Science and Welfare, Swedish University of Agricultural Sciences (SLU), 750 07 Uppsala, Sweden

**Keywords:** Drylands, Ecosystem restoration, Governance, Health, Social–ecological systems framework, Sustainability transformations

## Abstract

Drylands face urgent development challenges that are complex and interconnected, requiring holistic systems-based approaches. Here, we present a social–ecological systems framework to guide research, policy, and action across disciplines and sectors towards sustainability transformations in drylands for improved livelihoods and enhanced ecosystem, livestock, and human health. Our conceptual framework integrates three non-exclusive and complementary lenses of human-driven transformations: *systemic, structural* and *enabling/grassroots* change. Based on these three complementary lenses, along with three main phases of transformation and key associated actions, we briefly illustrate the framework’s value through the case of the Karamoja cross-border region of East Africa. We show how the framework can be used as an entry point to identify and analyse key challenges and their interlinkages; envision desired social–ecological systems; and explore pathways towards sustainability. The framework provides actionable guidance to co-develop context-specific interventions that support sustainability transformations in drylands.

**Supplementary Information:**

The online version contains supplementary material available at https://doi.org/10.1007/s13280-026-02389-9.

## Introduction

Drylands—hyper-arid, arid, semi-arid and dry sub-humid areas—cover about 41% of the Earth’s land surface (Safriel et al. 2005; Cherlet et al. 2018), an area which is projected to increase significantly due to climate change (Feng and Fu 2013; Huang et al. 2017). Besides their importance in terms of extent, drylands are critical as they harbour high biodiversity (Myers et al. 2000; Davies et al. 2012) and host an estimated 44% of the global cropland area, 50% of the world’s livestock, and 38% of the world’s population (Safriel et al. 2005; Cherlet et al. 2018). Drylands are characterised by high aridity levels, unpredictable rainfall, and degradation-sensitive soils, all of which limit primary production and livelihood opportunities. Population density, cropping, and urbanisation typically decline with increasing aridity (Safriel et al. 2005). In terms of land use, rangelands and croplands account for 90% of the global drylands area, and livestock-based livelihoods are prominent. While pastoralism dominates arid areas, agro-pastoralism is practised in semi-arid and dry sub-humid areas where rainfed agriculture is possible (Safriel et al. 2005; de Haan 2016). Large areas of the global drylands today suffer from land degradation (Zika and Erb 2009; Gibbs and Salmon 2015; Turner and Schlecht 2019; Vågen and Winowiecki 2019), which reduces the provision of ecosystem services (i.e. nature’s benefits to people) that are essential for human health and well-being, and accelerates global warming (IPBES 2018). Climate change is expected to increase not only overall aridity, but also the intensity and frequency of extreme events such as heatwaves, heavy rainfall, droughts, and floods (Arias et al. 2021; Seneviratne et al. 2021), exacerbating land degradation and biodiversity loss, and jeopardising human health and well-being (Haines et al. 2006; IPBES 2018; IPCC 2019).

About 90% of people in drylands live in low- and middle-income countries and lag significantly behind in terms of well-being and development indicators compared with those living in other areas (Adeel et al. 2005; Safriel et al. 2005). Dryland populations include some of the world’s poorest and most disadvantaged people (Middleton et al. 2011), making them particularly vulnerable to shocks and stresses (Sietz et al. 2011). Subsequently, food insecurity, malnutrition, and reliance on external aid are widespread problems in drylands (IPCC 2014; FAO et al. 2021). While population density remains typically low, many drylands are experiencing rapid population growth (MEA 2005; Cherlet et al. 2018), which amplifies the sustainability challenges that dryland populations face. Limited access to clean and safe drinking water is also a significant health issue in drylands. Prolonged water scarcity can lead to dehydration, poor hygiene, and increased waterborne diseases, such as cholera, dysentery, and hepatitis (UNICEF and WHO 2019; UNESCO and UN-Water 2020). In addition, access to healthcare services is often limited in remote dryland areas, exacerbating the impact of health issues by hindering early disease diagnosis and treatment (UNDP 2019).

A powerful force of change in drylands, as well as other landscapes inhabited by pastoralist communities worldwide, is land fragmentation resulting from land appropriation by governments and elites for conservation, privatisation of pastoralist commons, extraction of water, minerals, and oil, and urban expansion. Fragmentation has reshaped and continues to reshape pastoralist social–ecological systems, resulting in increased land scarcity, which in turn restricts seasonal livestock migration, reduces livestock production and constrains livelihood diversification (Reid et al. 2014; Lind et al. 2020a; Scoones et al. 2023). Drylands have historically tended to be both politically and economically marginalised through the remoteness of infrastructure, markets, industry, and state representation, resulting in a lack of economic investments as well as poor delivery of health and social services (Easdale and Domptail 2014; Jenet et al. 2016). However, the perception that drylands are marginal areas with little economic and political value is rapidly changing in many parts of the world. In East Africa, for example, the drylands are currently facing an unprecedented expansion of state and private sector investments in infrastructure, conservation, agricultural intensification, mining of high-demand minerals, and exploitation of fossil fuels, as well as ‘green’ energy (Mosley and Watson 2016; Lind et al. 2020a; Aalders et al. 2021). While such investments have the potential to trigger much-needed socio-economic development, they also risk increasing human pressures on dryland ecosystems and imposing restrictions on customary pastoralist livelihoods and ways of life.

While mobility has historically been a central strategy for accessing unpredictable and spatially and temporally variable resources in drylands (Turner and Schlecht 2019), ongoing land fragmentation (Cherlet et al. 2018) tends to hinder mobility and reduce resource access, particularly for the most marginalised in society. The multiple sustainability challenges facing the drylands evidently call for inclusive and effective governance across local, regional, national, and international scales. However, in many dryland regions, inappropriate policies by international, national, and sub-national actors that disregard the needs of (agro-) pastoralists continue to hamper the capacity of existing formal and customary institutions to govern dryland resources sustainably (Fratkin and Roth 2006).

All these challenges point to the critical and urgent need for dryland transformation. In contrast to processes of change that merely imply improved efficiency and functionality within existing dryland social–ecological structures, systems and relations, transformation signifies more radical and fundamental change, including system-wide changes in perspectives, structures and practices that address underlying causes of unsustainable human-nature relations (IPBES 2024b). Given the commitment of the United Nations Agenda 2030 for Sustainable Development to “*leave no one behind*” (UN 2015), drylands worldwide emerge as priority hotspots for action to achieve the Sustainable Development Goals (SDGs). Sustainable dryland development urgently requires identifying and enhancing synergies among food and nutrition security, ecosystem health, good environmental governance, poverty alleviation, and human health and well-being. Dryland environmental, social, and economic crises are deeply interlinked (IPBES 2024a). The multifaceted and intertwined nature of dryland challenges demands holistic, systems-based approaches to tackle them in a way that maximises co-benefits and brings about the transformative change needed to advance the 2030 Agenda and achieve its SDGs (Reynolds et al. 2007; Maestre et al. 2012; Liu et al. 2015; UN 2015; Whitmee et al. 2015; IPBES 2024b). Such transformative change requires multiple and diverse processes that, together, challenge unsustainable social–ecological relations at structural and systemic levels, as well as through mobilisation from below.

Social–ecological systems approaches recognise and emphasise the interconnectedness, interdependence, and dynamic interplay between the ecological (the biosphere) and human (economic, political, technological, and cultural) dimensions of the systems across different spatial and temporal scales (Folke et al. 2016; Biggs et al. 2021). When viewed through the lens of resilience theory (Folke et al. 2010), social–ecological systems approaches can serve as a powerful tool for understanding and addressing sustainability transformations (Folke et al. 2011; Moore et al. 2014; Olsson et al. 2014; Folke et al. 2016). However, social–ecological systems approaches and resilience theory have generally failed to incorporate and comprehensively address aspects of socio-political change that are central to transformations and key objects of study within the field of environmental social sciences (Stojanovic et al. 2016; Caniglia and Mayer 2021). More specifically, these aspects include the embeddedness of ecosystems within asymmetrical political, economic, and social relations of power; the importance of conflicts of interest, struggles and resistance for access to and control over natural resources; that solutions aimed at enhancing the resilience of social–ecological systems tend to have unintended and unequal outcomes for different social groups and individuals; and that natural resource management strategies often devalue or even disregard indigenous common property arrangements, livelihoods and knowledges (Osborne et al. 2021). Subsequently, we recognise that social–ecological systems are highly complex and power-laden and that the political contexts and power dynamics inherent in the transformations of these systems require recognition (Olsson et al. 2014; Osborne et al. 2021). Furthermore, we acknowledge that there are different ways of understanding and defining challenges facing social–ecological systems as well as multiple transformative pathways towards sustainability. Sustainable development and the persistence, adaptability, and transformability of social–ecological systems depend on navigating competing interests and power dynamics among multiple actors (Bennett et al. 2021). Because powerful actors tend to resist transformation and maintain the *status quo* (Cederlöf and Loftus 2023), overcoming entrenched power structures and relations is essential for enabling sustainability transformations for all. Challenging such unequal power dynamics requires ensuring that different stakeholders, in particular the most marginalised, have a voice in defining challenges and transformation pathways to sustainability (Osborne et al. 2021).

Here, we present a social–ecological systems framework designed to promote, facilitate, and guide research, dialogue, and action across disciplines and sectors towards sustainability transformations in drylands, aiming to simultaneously improve livelihoods and the health of ecosystems, livestock, and people. The framework has the ambition to complement the imperative to protect and conserve irreplaceable dryland ecosystem functions and services with the equally important need to reform political-economic structures that produce and uphold social inequality, and to provide conditions for people to democratically and inclusively engage in positive and productive processes of change. It can be used as an entry point to (a) identify and describe key challenges of the current social–ecological system and their interlinkages; (b) co-develop possible desired social–ecological system(s); and (c) identify pathways towards just and resilient sustainability transformations. Specifically, the paper aims to: (i) outline a social–ecological systems framework for sustainability transformations in drylands; (ii) illustrate its application based on a recent and comprehensive literature review, an analysis of secondary data, as well as transdisciplinary empirical research using the case of the Karamoja cross-border region in the drylands of East Africa; and (iii) discuss the framework’s implications, limitations, and potential transferability to other dryland regions.

## Conceptual framework: Sustainability transformations in drylands

Our conceptual framework is inspired by and builds on previous work and perspectives on social–ecological systems (Díaz et al. 2015; Masterson et al. 2019; Biggs et al. 2021), resilience thinking (Holling 1973; Folke et al. 2010), sustainability transformations (Moore et al. 2014; Olsson et al. 2014; Scoones et al. 2020), and sustainable development in drylands (Safriel et al. 2005; Blake et al. 2018). It is not intended to be an exhaustive or prescriptive model, but rather a generic entry point that highlights critical elements, relations, and processes. It is designed to stimulate integrative analysis and action in dryland contexts and can be adapted and expanded into more systematic, detailed, and context-sensitive models, providing a structured yet flexible tool.

### Framework development

This framework was developed as part of the research project *Drylands Transform* (2020–2025), which investigated the links between land health, livestock-based livelihoods, human well-being, land management, and governance in the drylands of East Africa. The project focused on the Karamoja cross-border region between Kenya and Uganda and aimed to generate knowledge that supports transformative change and sustainable development in drylands. The choice of Karamoja as the empirical focal point and case of sustainable dryland transformation was motivated by the following considerations: (i) it constitutes a historically and currently important dryland region from an ecological, social as well as economic perspective; (ii) it is a region faced by grave, complex, dynamic and urgent sustainability challenges; (iii) it is a region where diverse pastoralist communities share similar livelihood conditions while facing substantial differences in resource governance constraints and opportunities, depending on nationality, administrative organisation and local political realities. Developing this framework allowed us to integrate diverse disciplinary perspectives—spanning ecology, soil science, hydrology, agronomy, nutrition and public health, rangeland and livestock management, environmental and agricultural economics, development and governance studies, as well as political ecology—into a systematic and coherent interdisciplinary approach, ensuring that our work captures the complexity of dryland social–ecological systems. Co-developing the framework also provided a foundation for shared understanding of key concepts and approaches across disciplines, which is essential for effective interdisciplinary collaboration.

To build the framework, we combined insights from the scientific literature on social–ecological systems, resilience thinking, sustainability transformations, and dryland development (Holling 1973; Folke et al. 2010; Moore et al. 2014; Olsson et al. 2014; Díaz et al. 2015; Geels 2018; Masterson et al. 2019; Scoones et al. 2020; Biggs et al. 2021) with the expertise of our transdisciplinary team, who have extensive experience from working across various dryland contexts. We grounded the framework in the Karamoja cross-border region between Kenya and Uganda, drawing on primary quantitative and qualitative data collected within the *Drylands Transform* project on soil and land health, natural resource governance, food security and livelihoods, livestock management, and human nutrition and health (see e.g. Winowiecki et al. 2023; Bargués-Tobella et al. 2025; Drew and Knutsson 2025; Kawira et al. 2025; Kyanjo et al. 2025; Nabweteme et al. 2025; Schumann et al. 2025). These primary data were combined with secondary sources, including national survey datasets (e.g. Uganda Bureau of Statistics and Kenya National Bureau of Statistics), official statistics and reports from international organisations (e.g. FAO, WFP, UNDP), as well as relevant peer-reviewed and grey literature. Through an iterative process using the Karamoja case, we illustrated and refined the framework while maintaining its broad applicability to other dryland contexts.

### Framework structure and key elements

**Fig. 1:**
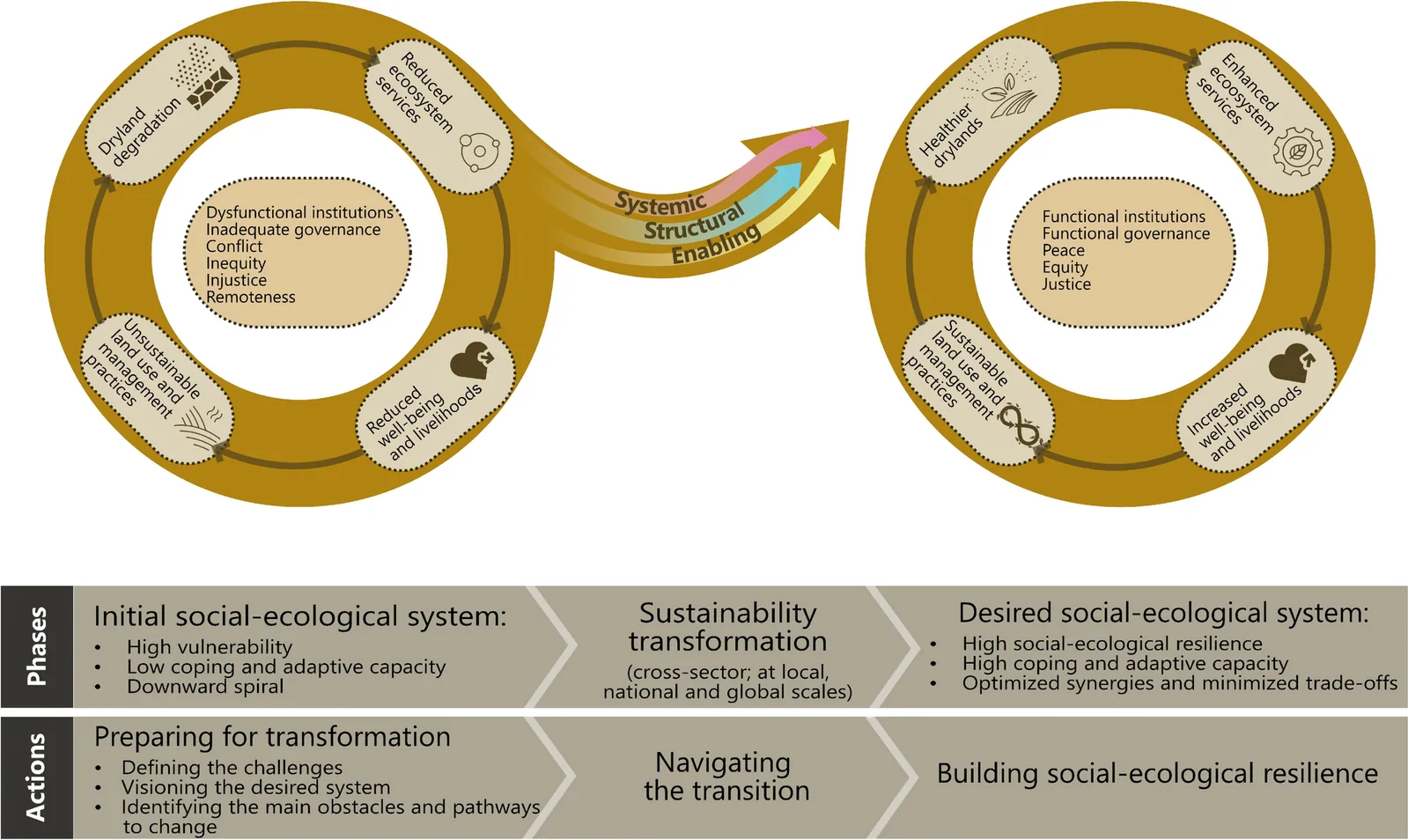
Conceptual framework of sustainability transformations in drylands, illustrating the three main phases of transformation (initial social–ecological system, sustainability transformation and desired social–ecological system) and the key actions associated with each phase. The left and right circles depict the initial and target/desired social–ecological systems, respectively, while the brown arrows within the circles illustrate key feedbacks. Institutions and governance systems are placed at the centre of the circles to highlight their central role in influencing all aspects of relationships within the social–ecological system. The arrow connecting the two circles represents the core transformation phase, and the three small arrows within, the three lenses of transformation (i.e. *systemic, structural* and *enabling/grassroots*). Exemplary transformation pathways include the restoration of degraded drylands and the implementation of more functional and inclusive governance arrangements

The framework (Fig. 1) is structured around a general transformation process that moves from an initial social–ecological system facing substantial sustainability challenges towards a system characterised by resilient and just social–ecological relations via multiple, parallel processes of change. The framework furthermore describes three key actions along this transformation process: (i) preparing for transformation; (ii) navigating the transition; and (iii) building social–ecological resilience in the transformed system (Olsson et al. 2014). The first phase, preparing for transformation, involves three key steps that form the core of the framework’s practical application: (i) defining the challenges of the initial social–ecological system, (ii) visioning the desired social–ecological system, and (iii) identifying and operationalising pathways towards sustainability (see Box 1). The left and right circles depict the initial and desired social–ecological systems, respectively, while the arrow connecting them illustrates the core transformation phase or transition. Within each circle, arrows illustrate key feedbacks that maintain the current system or reinforce system dynamics. At the centre of each circle, key social dimensions of the system (such as institutions, governance, peace/conflict, justice/injustice, and equity/inequity) are highlighted to underscore their central role in shaping the dynamics and functioning of the social–ecological system.

**Box 1:** Key steps for using the framework

• *Step 1* Defining the challenges of the initial social–ecological system (Fig. 1, left circle) Co-define the main challenges in the current social–ecological system. Examine the key feedbacks along the “land degradation → reduced ecosystem services → reduced well-being and livelihoods → unsustainable land management” loop. Consider how social dimensions (institutions, governance, equity, etc.) both influence and are influenced by these dynamics. When gathering data—whether from existing sources or through new collection—consider approaches that capture the core social and ecological dimensions of the system, as well as the key interactions and feedbacks between and across them. This is particularly important when designing new data collection to ensure that the system’s dynamics are adequately represented	
• *Step 2* Visioning the desired social–ecological system (Fig. 1, right circle) Co-develop visions of the future target system. Scenario development and evaluation approaches can support this process. Reflect on the key feedbacks along the proposed “improved dryland health → enhanced ecosystem services → improved well-being and livelihoods → sustainable land management” loop. Consider how governance of natural resources through various formal and informal institutions, along with efforts to improve equity, justice, and peace, can interact with and reinforce ecological and livelihood improvements. Include diverse actors and voices, and draw on multiple knowledge systems, to ensure the envisioned system reflects a broad range of perspectives	
• *Step 3* Identifying and operationalising pathways to change Co-explore with different stakeholders/actors potential pathways to move the system from its current state towards the desired social–ecological system. Apply the three lenses—systemic, structural, and enabling/grassroots—to understand how to make different pathways possible. Identify obstacles and design actionable strategies. Pathways may, for example, involve ecological restoration, governance reforms, or strengthening local agency. Translate promising pathways into context-specific interventions. The visions and scenarios co-developed in Step 2 guide this process, ensuring pathways are relevant to alternative envisioned futures and local priorities	

Following Scoones et al. (2020), we approach human-driven sustainability transformations of social–ecological systems using three distinct but complementary lenses: *systemic, structural and enabling/grassroots* change. The different lenses highlight the need for multiple perspectives on the drivers and potential obstacles of transformations towards sustainability. In Fig. 1, these lenses are represented by the three smaller arrows within the main arrow connecting the two circles, which depicts the core transformation phase. When viewing sustainability transformations through a *systemic* lens, attention is directed to key interdependencies between ecological and social components within a given system. A particular focus is set on feedbacks that either reinforce (positive feedback) or dampen (negative feedback) system dynamics over time, and how changes in one part of a social–ecological system, in pursuit of sustainable transformations, can affect other parts. At the core of our framework, the systemic lens directs analysis toward key system elements and their interdependencies, highlighting two primary positive feedback loops: one illustrating how the current social–ecological system can spiral downward (Fig. 1, left circle), and another showing how the desired system can build long-term social–ecological resilience (Fig. 1, right circle). Epistemologically, a systemic lens provides a functionalistic perspective on transformation, focusing on how interactions and feedbacks can be steered to improve system functioning (albeit with an acknowledgement of uncertainty, the propensity for nonlinear responses, and the inherent complexity of system interactions). By contrast, applying a *structural* lens to sustainability transformations shifts the focus to the underlying foundations of political, economic, and social relations that underpin unsustainable resource practices and unequal and unfair control over natural resources. We acknowledge that functional understandings of social–ecological systems based on systems theory often incorporate complex relations between structure and function (see e.g. Young 2012). However, following Scoones et al. (2020), we find it analytically useful to distinguish between recognising the need for changes in social–ecological system functions and recognising the need for changes in the way economic “production and consumption are organised by societies”. Epistemologically, a structural lens to sustainability transformations draws on critical social science theories that focus on how particular arrangements of economic, political and social relations influence the distribution and use of rights to natural resources, as well as the management of ecosystems (Robbins 2019). Complementing the focus on desired configurations of the system’s fundamental structures and the management of system dynamics, an *enabling/grassroots* lens to sustainability transformations emphasises the promotion of social attributes, or capacities, that empower individuals and communities to act on their own behalf. Enabling sustainability transformation involves creating the conditions for agency—the deliberate exercise of individual or collective will—with particular attention paid to the interests of the most excluded. By strengthening agency, communities can actively engage in decision-making, innovate locally appropriate solutions, and influence governance and resource management, thereby contributing directly to sustainability transformations. Epistemologically, an enabling lens combines social science theory on collective action and agency with social–ecological systems theory on self-organisation, highlighting the human capacities and knowledge needed to manage uncertainty and adapt to social–ecological shocks and stresses. Together, these three lenses for sustainability transformations strongly and principally resonate with prominent attempts to conceptualise the socio-technical requirements for transitions to more sustainable resource use and consumption practices (e.g. Geels 2018).

We see these three lenses as analytically distinct yet empirically highly complementary and interdependent. Drawing on well-established, multidisciplinary scholarly work on social–ecological change, they offer a conceptual checklist of aspects of transformation towards sustainability that need careful attention. As such, we propose that the three lenses be applied in parallel to any specific transformation context. Together, they help users think through and structure potential transformation pathways and interventions, identifying opportunities to influence social–ecological dynamics in ways that support more sustainable and equitable outcomes. For example, what are the economic structures that reinforce unsustainable and unfair system dynamics and community natural resource practices, and how can such structures be changed or reformed? How can communities mobilise around more sustainable natural resource use and management practices to enhance ecosystem functions and services? And how can such mobilisation contribute towards and/or be enabled by reformed economic, political or social structures? By explicitly combining *structural* and *enabling/grassroots* perspectives with our core *systemic* approach, the framework encourages users to consider power relations, local agency, conflicts of interest, equity, and the multiple ways challenges and transformation pathways can be defined and pursued. Such factors are often overlooked in conventional systems-focused approaches, as noted in the ‘Introduction’, and risk being ignored or overlooked without such an explicit focus.

The initial system we depict (Fig. 1, left circle) synthesises the current state of the social–ecological system in many drylands today, highlighting key challenges and the feedbacks that reinforce them: The degradation of dryland ecosystems reduces the provision of ecosystem services, which in turn negatively affects people’s livelihoods and well-being. Poverty and livelihood insecurity can force people to migrate, trigger conflicts, and diminish ecosystem stewardship, resulting in unsustainable land use and management practices that exacerbate land degradation. Additionally, dysfunctional institutions and governance systems at national, sub-national, and local levels, alongside the exploitation of drylands by state and non-state actors, often lead to inequitable access to and distribution of ecosystem services and do not support sustainable development and stewardship. This has major consequences for land degradation and conflict. Such a downward spiral maintains the system in an unsustainable social–ecological trap with low coping and adaptive capacity and a high degree of vulnerability to shocks and stresses. These shocks and stresses can be biophysical, socio-economic, and political, including climate and environmental change, drought, extreme heat, floods, violent conflict, political instability, or increased pressure on land and water resources.

The transformation we envisage involves a profound reorganisation of the current social–ecological system to attain more sustainable and equitable outcomes (Olsson et al. 2014; Biggs et al. 2021). In the desired/target system (Fig. 1, right circle), healthy dryland ecosystems support the provision of ecosystem services, leading to enhanced livelihoods and well-being, which can promote ecosystem stewardship and more sustainable land use and management practices. Well-functioning institutions and inclusive governance systems at national and sub-national levels that prioritise the needs and rights of rural communities and work to strengthen customary systems of governance can enable and support ecosystem stewardship, peace, equity, and justice, and ensure fairer access to ecosystem services. Here, positive feedback loops contribute to building long-term social–ecological resilience, defined as “the capacity to adapt or transform in the face of change in social–ecological systems, particularly unexpected change, in ways that continue to support human well-being” (Folke et al. 2016). In contrast to the initial social–ecological system, this system optimises synergies and minimises trade-offs among multiple SDGs, in particular between food security (SDG 2), restoration of degraded land and ecosystem health (SDG 15), climate action (SDG 13), water security (SDG 6), environmental governance (SDG 16), poverty alleviation (SDG 1), and improved health (SDG 3) and equity (SDGs 5).

Building on recent attempts to conceptualise transformational social–ecological change (Olsson et al. 2014; Geels 2018; Scoones et al. 2020; IPBES 2024b), our framework integrates insights from systems and resilience theory with well-established social science understandings of the central role of societal structures and human agency in shaping social–ecological change. While Scoones et al. (2020) acknowledge the importance of systemic, structural, and enabling/grassroots perspectives as alternative and epistemologically distinct understandings of social-ecological change, our framework advances beyond this by combining these three lenses within a structured, forward-looking three-phase transformation (Olsson et al. 2014) framework to guide sustainability transformations. Additionally, the framework is explicitly designed for dryland social–ecological systems.

We propose that our framework, through a conscious, systematic, and explicit integration of the three lenses, offers a more comprehensive and coherent approach to the identification and operationalisation of genuinely transformative pathways towards sustainability (as opposed to mere improvements in social–ecological functionality and effectiveness). From this, a set of diagnostic propositions follows: (i) Human-driven sustainability transformations of dryland social–ecological systems are unlikely when interventions focus primarily on a single analytical dimension, for example systemic dynamics, without simultaneously engaging with the other dimensions of structural change and enabling conditions for local agency; (ii) Transformation pathways that consciously combine systemic interventions with structural reforms and efforts to strengthen local agency are more likely to generate durable and equitable sustainability transformations than approaches that address these dimensions in isolation; (iii) Applying the framework across different dryland contexts is likely to reveal divergent transformation pathways, reflecting variations in governance arrangements, power relations, visions of desired social–ecological systems, and ecological conditions.

### Using the framework

The framework can guide multiple users—including researchers, non-governmental organisations (NGOs), local governments, and community-based organisations—in analysing, envisioning, and planning sustainability transformations in drylands. To support such use, Box 1 outlines three steps for applying the framework in practice when preparing for transformations, the first phase of our framework (Fig. 1). While Fig. 1 depicts the full, forward-looking transformation process, including phases that unfold over time, the practical guidance provided here focuses on the “preparing for transformation” phase, for which Box 1 details the three key steps. These steps are not prescriptive but rather a flexible guide that can be adapted to different contexts, data availability, and stakeholder needs. More nuanced insights into the framework’s operationalisation and lessons learned from its application in the Karamoja case are presented in sections ‘Applying the framework: The case of the Karamoja cluster in the drylands of East Africa’ and ‘Concluding discussion’.

Applying these steps in practice does not require comprehensive datasets from the outset. In particular, the first step (defining the challenges of the initial social–ecological system) relies on an initial system diagnosis that can be informed by a limited set of core data and other information sources. At a minimum, this includes information on (i) key ecological conditions and dynamics (e.g. land health, water availability, climate variability), (ii) livelihoods, well-being, and resource-use practices of different social groups, and (iii) institutional arrangements, governance structures, and power relations shaping access to and control over natural resources. Such diagnostics can often be initiated using existing secondary sources (e.g. remote sensing products, census and household survey data, official statistics, reports, policy documents, and previous studies), complemented by local knowledge, and targeted primary data collection when critical gaps are identified. Together, these sources typically provide a combination of quantitative and qualitative information needed to characterise key social and ecological system components, as well as the interactions and feedbacks that shape current system dynamics (Biggs et al. 2021). Rather than aiming for comprehensive coverage, the framework is intended to help structure available information, identify key feedbacks and uncertainties, and prioritise data and monitoring needs to support learning and adaptive management. In contrast, the subsequent steps—visioning the desired social–ecological system (Step 2) and identifying and operationalising pathways to change (Step 3)—rely more strongly on structured participatory processes and deliberation, including scenario development exercises and/or other established participatory methods (Pereira et al. 2018), than on detailed system diagnostic data. Existing long-term visions and agendas (e.g. international agendas such as Agenda 2030, the International Year of Rangelands and Pastoralists (IYRP) 2026, the UN Decade on Ecosystem Restoration 2021–2030, or the’30 by 30’ nature conservation initiative), as well as national or regional development or environmental strategies and plans (e.g. Kenya Vision 2030; Ethiopian National Drylands Restoration Strategy, Developed Rajasthan @2047 Vision Document), can provide valuable inputs for these steps.

The steps are inspired by a back-casting approach to sustainability (Robinson et al. 2011; Vergragt and Quist 2011), in which a desired future state is used to inform present-day analysis and planning. In our application, however, the criteria for sustainability are defined normatively in relation to the SDGs rather than through an explicit step of criteria-setting as in classical back-casting. Table 1 provides a structured overview of how this back-casting logic can be operationalised during the “preparing for transformation” phase.

**Table 1:** Examples of typical inputs, key actions, and intermediate outputs associated with the three steps for applying the framework during the “preparing for transformation” phase

Step	Inputs	Key actions	Outputs
*Step 1* Defining the challenges of the initial social–ecological system	Existing and newly collected ecological and social data; local and indigenous knowledge; key stakeholder perspectives	Co-define challenges; analyse social–ecological interactions and feedbacks; synthesise available information	Shared diagnosis of system dynamics, key challenges, feedbacks, and constraints on sustainability transformations
*Step 2* Visioning the desired social–ecological system	Shared system diagnosis from Step 1; existing visions and plans; initial stakeholder priorities, interests, and perspectives	Facilitate participatory visioning, including through scenario development approaches; include diverse actors, voices, and knowledge systems	Co-developed visions of desired social–ecological systems
*Step 3* Identifying and operationalising pathways to change	Shared system diagnosis from Step 1 and co-developed visions of desired social–ecological systems from Step 2	Identify and assess potential transformation pathways using the systemic, structural, and enabling/grassroots lenses; explore trade-offs, obstacles, and synergies; co-design actionable strategies and interventions	Context-specific transformation pathways and portfolios of interventions

To further support operationalisation, Table 2 provides illustrative example indicators to guide the application of the three lenses during the “preparing for transformation” phase and to inform learning and adaptation as transformations unfold.

**Table 2:** Illustrative example indicators aligned with the three lenses to support application of the framework

Lens	Indicators
Systemic	Condition and trends of key ecosystem components (e.g. soil health, vegetation cover, water availability); livelihood security and diversity; evidence of reinforcing or dampening social–ecological feedbacks (e.g. between land use practices, ecosystem services, and well-being)
Structural	Land tenure and access arrangements, including the degree of security and equity; governance institutions and policies that shape resource use and benefit distribution; concentration or distribution of decision-making power; market access and economic conditions that influence livelihoods and land management
Enabling/grassroots	Opportunities for participation in decision-making, particularly for marginalised and less powerful groups; existence and strength of collective action and local organisations (e.g. community-based organisations, producer groups, customary institutions); capacities for learning, innovation, and adaptive action (e.g. skills, social networks, and organisational arrangements) at individual and community levels

Although the three steps are presented sequentially, in practice, they overlap and iterate, relying on continuous cycles of reflection, action, and learning. The framework, therefore, embeds an adaptive management logic (diagnose, co-design, implement, and co-learn). During the phase of preparation for transformation, this adaptive approach supports participatory analysis of the initial social–ecological system, visioning, and co-design and implementation of pathways. Once a desired social–ecological system has been approached, adaptive management focuses on sustaining and reinforcing the system through continuous learning and actions that contribute to its resilience, which constitutes the third action of our framework (Fig. 1). Drawing on the general principles outlined by Biggs et al. (2015), this includes maintaining diversity and redundancy, managing connectivity and feedbacks, fostering complex adaptive systems thinking, encouraging learning, broadening participation, and promoting polycentric governance. Building resilience thus entails institutionalising and routinising the changes promoted by transformations, enhancing the capacities of individuals, communities and other actors to act on their own behalf, and strengthening desirable social–ecological feedbacks while maintaining the system’s adaptive and transformative capacity to respond to future challenges (Folke et al. 2011). Responsibility for evaluating and maintaining a resilient social–ecological system is, by necessity, polycentric and rests with all actors across scales. Collaboration among actors must continue to ensure that powerful actors do not dictate the dynamics of the resulting social–ecological system, and to promote human-environment relations and institutional mechanisms that are sustainable for different social groups and the environment.

## Applying the framework: The case of the Karamoja cluster in the drylands of East Africa

In this section, we illustrate the use of the proposed framework through its application to the Karamoja cluster cross-border area in the East African drylands, with an emphasis on Kenya and Uganda. As outlined in the previous section (Box 1), the three key steps in preparing for transformation involve (1) defining the challenges of the initial social–ecological system, (2) visioning the desired social–ecological system, and (3) identifying and operationalising pathways to change. The Karamoja cluster case was central to the framework’s development and provides valuable context for demonstrating how these steps can be implemented in practice. We note that this illustration reflects only the activities and data available from the *Drylands Transform* project to date. However, as the application of the framework is inherently iterative and adaptive, this example provides a partial demonstration of how the steps can be implemented in practice rather than a complete or final application.

### Defining the challenges of the initial social–ecological system

#### The Karamoja cluster

**Fig. 2:**
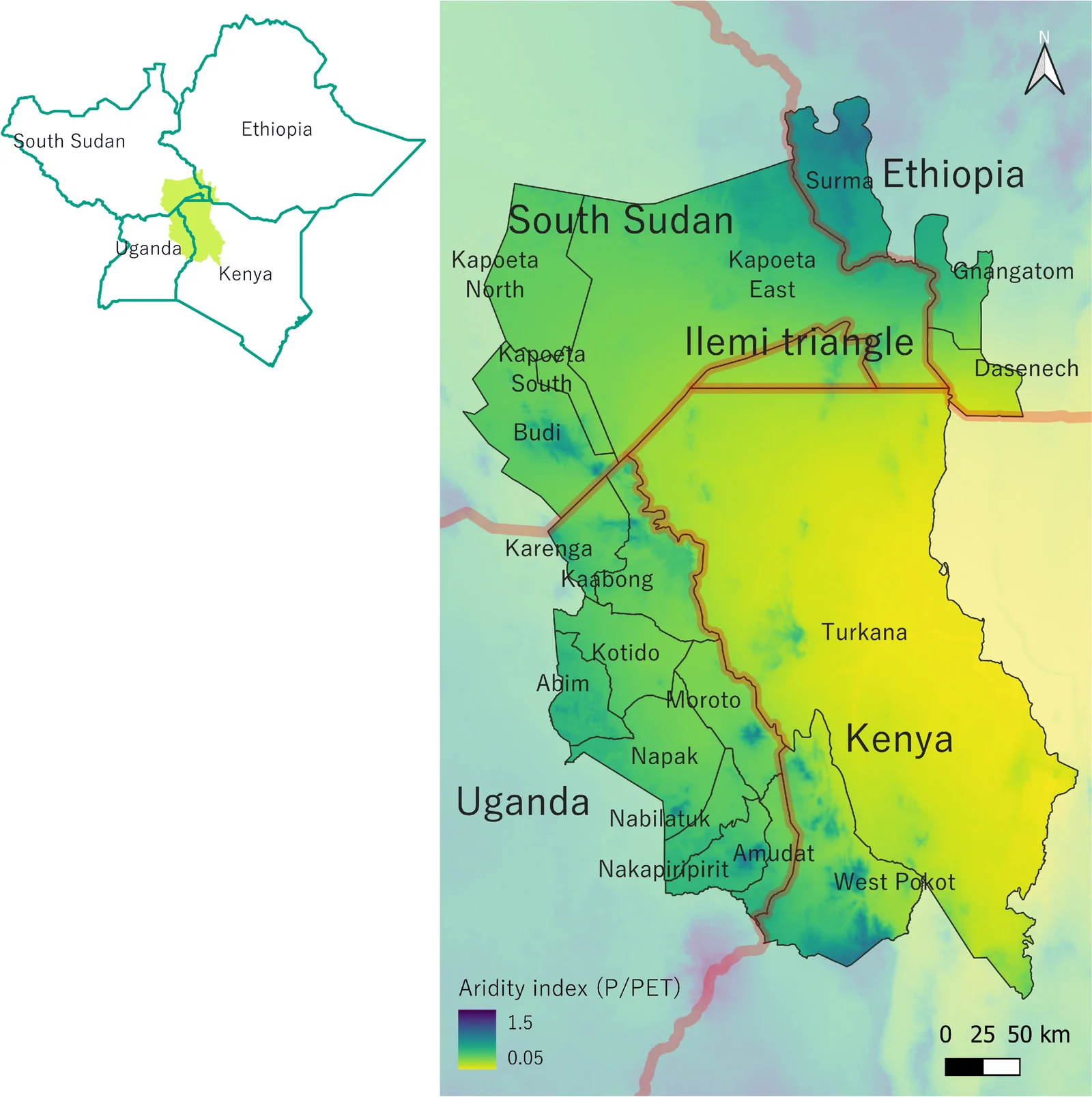
Map of the Karamoja cluster as defined by the Intergovernmental Authority on Development (IGAD 2022), indicating the names of the districts/counties/woredas. The background colour indicates the mean aridity index (AI), defined as the ratio of mean annual precipitation (P) to mean annual potential evapotranspiration (PET) (UNEP 1992), derived from the ‘Global Aridity Index and Potential Evaporation Database’ for the period 1970–2000 (Zomer et al. 2022). Aridity index values between 0.05 and 0.20 correspond to an arid climate, between 0.20 and 0.50 to a semi-arid climate, between 0.5 and 0.65 to a dry sub-humid climate, and above 0.65 to humid. Note that AI values are indicative and subject to uncertainty arising from spatial and temporal variability in the underlying climate data

The Karamoja cluster is defined by the Intergovernmental Authority on Development (IGAD) as the cross-border region encompassing the Karamoja sub-region of Uganda (Kotido, Kaabong, Karenga, Nabilatuk, Abim, Moroto, Napak, Amudat and Nakapiripirit districts), the West Pokot and Turkana counties of Kenya, the Budi, Kapoeta North, Kapoeta South, and Kapoeta East counties of South Sudan, and the Dasenech, Gnangatom, and Surma woredas of Ethiopia (Fig. 2). The cluster also includes the Ilemi Triangle, a disputed area of land between Ethiopia, South Sudan and Kenya. Most of the Karamoja cluster is classified as drylands (UNEP 1992) except for some areas at higher altitudes (Fig. 2).

The Karamoja cluster is home to several pastoralist and agro-pastoralist communities, many of whom are closely related and share similar languages, cultures and ways of life (IGAD 2022). At the same time, the region is characterised by a high degree of diversity in terms of ethnic groups, rainfall regimes, land uses, livestock species, cropping systems, and natural resource governance mechanisms and regimes. Livestock keeping is the primary livelihood option in the Karamoja cluster (Powell 2010) and pastoralist communities often move with their livestock across national and international administrative borders in search of pasture and water. Transhumance, the most common form of migration, involves the seasonal movement of part of the family (primarily young men led by elders and adult men or household heads) with their livestock.

**Fig. 3:**
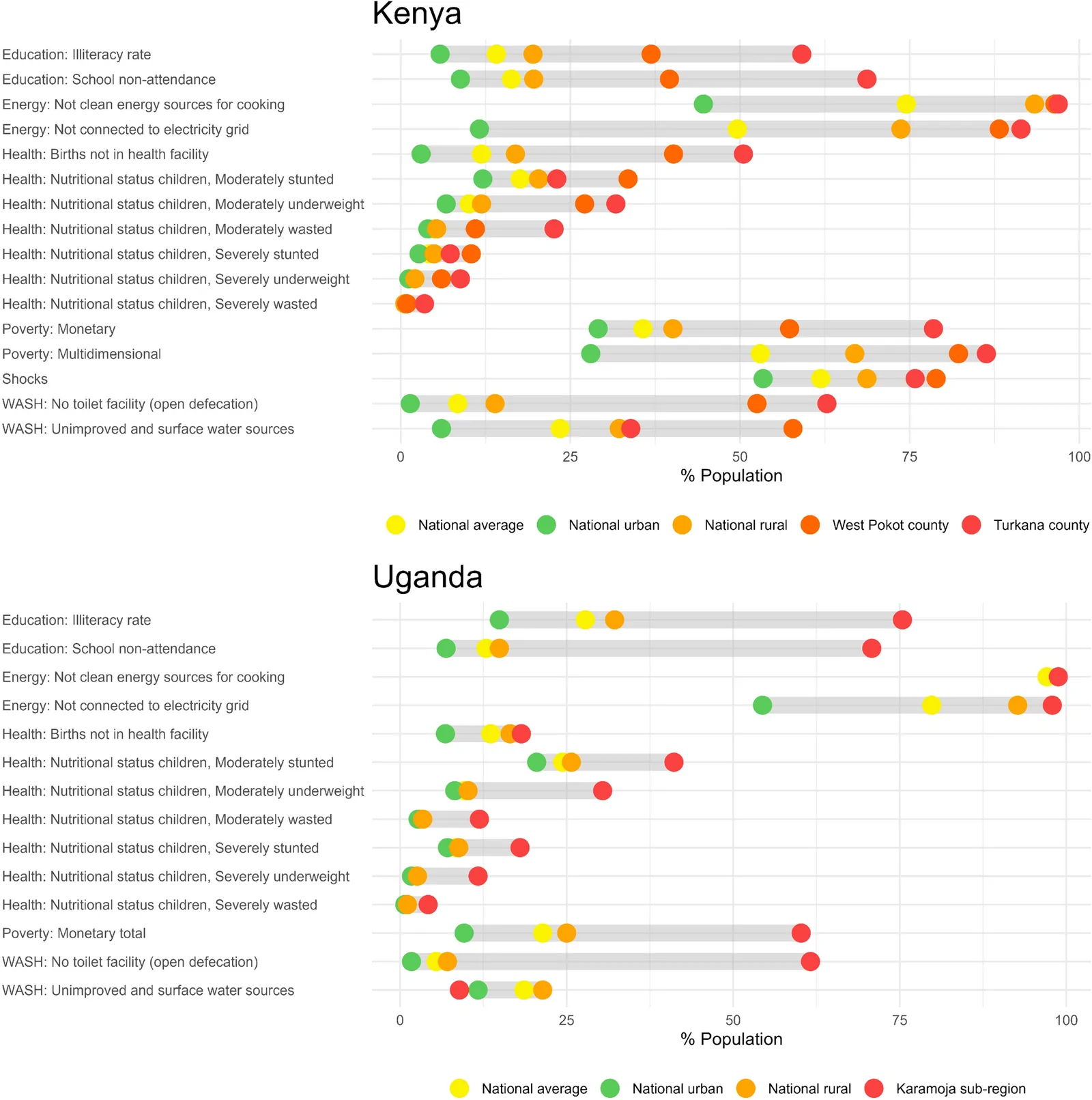
Key development and well-being indicators in the Karamoja cluster areas of Kenya and Uganda (i.e. West Pokot and Turkana counties of Kenya, and the Karamoja sub-region of Uganda) as compared to the respective national averages of Kenya (upper panel) and Uganda (lower panel). The Karamoja cluster is defined by IGAD as the cross-border region encompassing the Karamoja sub-region of Uganda, the West Pokot and Turkana counties of Kenya, the Budi, Kapoeta North, Kapoeta South, and Kapoeta East counties of South Sudan, and the Dasenech, Gnangatom, and Surma woredas of Ethiopia. Only the Kenya and Uganda parts of the cluster are included here. Data on the different indicators were compiled from the most recent available national surveys (2014–2023). All values are expressed as percentages of the relevant population. A detailed description of each indicator, the raw data, and the references to the data source are provided in Supplementary Tables S1 and S2. Note that the underlying datasets originate from different surveys conducted at different points in time

Like many drylands across the world, the Karamoja cluster faces a range of interlinked challenges, including widespread poverty, food and nutrition insecurity, violent conflict, socio-economic and political marginalisation, and recurring climate-related shocks, such as droughts and floods (FAO et al. 2023) and lags significantly behind in terms of development and well-being indicators. For example, in Kenya and Uganda, areas within the Karamoja cluster consistently perform poorly on education, energy security, health, poverty, and water, sanitation, and hygiene (WASH) indicators compared to the national average values (Fig. 3; see Supplementary Tables S1 and S2 for indicator definitions, data sources, and underlying values).

In recent decades, there have been numerous interventions by governments, NGOs, and research projects in this area, but the improvements have been insufficient (Lind et al. 2016; Muhereza 2017; Akall 2021; Caravani 2024). This lack of progress has been widely attributed to prevalent siloed approaches and disconnected actions and policies that treat different challenges and elements of the social–ecological systems in isolation (Barrantes and Caravani 2020). The rationale for the persistent belief that more intensive and sedentary agricultural production is the primary solution to food and income insecurity has also been questioned. Trends towards increasingly unpredictable rainfall amplify the challenge of unsustainable agriculture. The lack of development continues to harm the feelings of self-worth and identity in the population, which are essential for resilient, peaceful societies. Here, we describe three key interlinked challenges faced in the Karamoja cluster using our framework (Fig. 1): (a) land degradation; (b) inadequate governance; and (c) unsustainable livelihoods and food insecurity.

#### Land degradation

Land degradation—defined here as a negative trend in land health/condition caused by direct or indirect human-induced processes, and expressed as the long-term decline or loss of biodiversity, ecosystem functions, or their benefits to people in any terrestrial and associated aquatic ecosystems (IPBES 2018; IPCC 2019)—is a major concern in the Karamoja cluster, where a large proportion of people’s livelihoods heavily depend on land ecosystem services. Widespread land fragmentation, land-use changes, restricted mobility, and poor land management have been widely documented as contributing to land degradation in the form of soil erosion, woody plant encroachment and invasive species, and declines in plant species diversity and biomass, soil health, and overall land productivity (Maitima et al. 2009; Ng et al. 2016; Karamage et al. 2017; Mganga 2022).

Results from systematic assessments of soil and land health conducted using the Land Degradation Surveillance Framework (LDSF) (Vågen and Winowiecki 2023) in four 10 × 10 km sites across the Karamoja cluster—two in Kenya (Turkana and West Pokot counties) and two in Uganda (Napak and Moroto districts)—indicate contrasting degradation dynamics both across and within sites (Winowiecki et al. 2023; Bargués-Tobella et al. 2025). Soil erosion was a major issue at the Kenyan sites, where severe soil erosion in the form of gully and rill erosion was detected in 50–100% of sampled plots per cluster (10 plots per cluster, 16 clusters per site). In contrast, erosion prevalence was much lower at the Ugandan sites, affecting less than 10% of plots per cluster. In the Turkana site, woody plant encroachment was evident, with an average woody plant density of 275 individuals ha^−1^ (compared with only 18 individuals ha^−1^ in the Napak site) and nearly half of the sampled trees belonging to *Acacia reficiens* Wawra*.* The site in West Pokot showed severe erosion despite no signs of woody encroachment, yet exhibited high diversity of tree species, several of which are important for fodder, food and fuelwood (Swärd 2023). Soil infiltration capacity was relatively low across all sites, with median values ranging from 10 to 60 mm h^−1^, and showed a clear negative relationship with erosion prevalence. These assessments provide a robust baseline of land health conditions across sites, but do not yet capture temporal trends in degradation or recovery across broader areas. Field data, such as those from the LDSF, have been successfully combined with Earth Observation data to enable monitoring of spatial and temporal changes in land health at broader scales, as demonstrated in other dryland regions (e.g. Vågen and Winowiecki 2019; Allred et al. 2025). Such monitoring is critical for tracking land health dynamics over time, improving understanding of degradation processes and feedbacks, and supporting learning and adaptive management in the Karamoja cluster.

The deterioration of land health involves a decrease in land ecosystems’ functions and related ecosystem services such as flood regulation and food production. Feedback loops across the plant-soil–water nexus may further amplify land degradation processes. For example, reduced soil health and hydrological functioning lead to increased surface runoff, reduced water available to plants, and a higher risk of erosion and flooding. This spiral of land degradation might continue and eventually reach an extreme state of land degradation where land becomes largely unproductive. While these feedbacks are well established conceptually, their strength and thresholds vary across contexts and remain insufficiently quantified for much of the Karamoja cluster, highlighting the importance of sustained monitoring.

Land degradation in the Karamoja cluster has been attributed to a combination of direct and indirect drivers, including anthropogenic climate change with more extreme events such as floods and droughts, overgrazing, cropland expansion, poor agricultural practices, unsustainable tree harvesting (e.g. for charcoal production), mining, increased pressure on land, inadequate governance at national and sub-national levels, and a decline in traditional resource management institutions and governance structures that enforce norms and values for the sustainable use of land (Inselman 2003; Omondi and Odhiambo 2009; Mganga 2022). Nakalembe et al. (2017) estimate an almost threefold increase in cropland area in the Karamoja sub-region of Uganda between 2000 and 2011. The expansion of cropland, mining, infrastructure, and human settlements has reduced pastoralist mobility and increased pressure on the remaining rangelands, exacerbating rangeland degradation (Enns 2019). Although these drivers have been widely documented, their relative importance, interactions, and spatial distribution remain uncertain. Because they interact and vary in importance across space and time, monitoring is critical for better understanding and disentangling the drivers of land degradation.

Land degradation, in turn, reduces the capacity of land ecosystems to support forage, livestock, and crop production, as well as the provision of other critical ecosystem services such as water regulation, with adverse impacts on livestock health and (agro-)pastoralists’ food security, livelihoods, health, and overall well-being (IPBES 2018). However, in the Karamoja cluster, empirical evidence linking specific forms and degrees of land degradation to human health and well-being outcomes has not been systematically assessed, highlighting an important knowledge gap and the need for integrated research that links land health, livelihoods, and human health and well-being.

#### Inadequate governance: Conflict, land reform and inequality

Since colonial times, pastoral communities in the Karamoja cluster have experienced continuous political and economic marginalisation, as evidenced by their under-representation in national, regional and international policymaking forums (Pavanello 2009). While customary institutions continue to play a significant role in governing livelihoods, including conflict resolution and resource access, formal policies, decision-making, and actions by state and non-state actors at different levels have tended not only to undermine these institutions but also to be inadequate in responding to the priorities and needs of the diverse pastoralist communities in the region (Flintan et al. 2021). While the governance inadequacies outlined below are well supported by both existing literature and recently collected primary data, important gaps and uncertainties remain. More specifically, both our and other previous research on land governance in the Karamoja cluster tend to rely rather heavily on the experiences and perspectives of local communities, authorities, and organisations, and less on the perspectives and priorities of national governments and private sector investors (often because this information is difficult to access). This implies that information on important events, plans, and decisions relevant to natural resource governance is characterised by bias and uncertainty.

Across the cluster, conflict between pastoralist groups has constituted an enduring governance challenge for both state and non-state actors. One of the few available quantitative studies shows that during 2000–2013, the Karamoja cluster region experienced 325 violent conflict events (Ayana et al. 2016). Results from more than 200 qualitative interviews conducted between 2022 and 2024, complemented by recurrent focus group interviews and scenario workshops in four focus study sites in Kenya and Uganda (Napak and Moroto districts, and Turkana and West Pokot counties), show that violent conflict between ethnic pastoralist groups is both perceived and experienced as a key disruptive and destructive force that prevents long-term livelihood sustainability in all four districts/counties (Drew and Knutsson 2025). However, while Napak and Moroto are sites of recent events of violent conflicts, inhabitants of West Pokot and Turkana predominantly experience conflict when migrating livestock into Uganda or other counties in Kenya. Due to the enduring prevalence of inter-ethnic conflict in Kenya and Uganda, regional and state interventions in the Karamoja drylands have, therefore, often focused on issues of insecurity, as pastoralist populations have been perceived and framed as violent and, consequently, a threat to national security (e.g. Czuba 2019; Kilaka 2022). Mitigating conflict in the East African drylands, however, has proven complex, particularly because the drivers of conflict remain debated and dynamically changing over time. While conflict event data provide useful quantitative signals, they offer limited insight into underlying causal mechanisms, which remain context-specific and difficult to disentangle empirically. Often cited factors that trigger present dryland conflicts in the region include the proliferation of arms as a major cause of violence (e.g. see Fukui and Markakis 1994; Mkutu 2008), commercialisation of livestock theft and political-economic decentralisation (Green 2008; Schlee and Shongolo 2012; Greiner 2013; Carrier and Kochore 2014; Meyerson 2023), and communal land annexation and fragmentation (Mamdani 1982; Drew 2020). In both Kenya and Uganda, the national government has at times attempted to come to terms with outbreaks of conflict by ‘pacifying’ pastoralists using repressive methods. Our qualitative data collected in Napak and Moroto include numerous testimonies of the negative impacts of harsh measures by the Ugandan Defence Forces (UDF) to seize and control firearms as part of recurrent disarmament programmes since the early 2000s. In addition, during the whole fieldwork period, the presence of the Ugandan Defence Forces (UDF) was continuously visible through its regular patrols, roadblocks and missions to retrieve stolen livestock. Overall, state-sponsored attempts to control conflict and violence often entail severe restrictions on livestock migration for water and pasture, and also negatively impact the capacity of customary institutions to manage conflict and resource access (Czuba 2019).

Another enduring governance challenge relates to pastoralist land resources. Sedentarisation, formalisation of tenure institutions through land titling, and commercial forms of herding and agriculture all have been and continue to be advocated for by states, many NGOs, and some academic scholars as a solution to land degradation and limited development in terms of access to education, health care, etc. (Fig. 3). For example, land acts have triggered large areas of communal land in southwestern West Pokot to be settled and demarcated into individual plots with a focus on ‘modern’ forms of agriculture with the ambition to restore degraded land and increase production (Republic of Kenya 2012b; Drew and Knutsson 2025). Other sections of communal land in this area have already undergone group titling (Republic of Kenya 2012a), and the recent implementation of the Community Land Act (CLA) (Republic of Kenya 2016) has accelerated processes of land intensification, commodification, sedentarisation and demarcation. In Turkana, where land titling and sedentarisation have been less pronounced, the introduction of the CLA has also started to change existing land tenure regimes, albeit not to the same degree as in West Pokot. In the Karamoja sub-region of Uganda, post-disarmament peace has enabled state and non-state actors to intensify the implementation of schemes promoting settled agriculture (e.g. Republic of Uganda 2000). Whilst our qualitative interviews show that some pastoralist groups and individuals across all four studied districts/counties call for and benefit from increasing sedentarisation, individualisation and formalisation of land tenure, they also reveal frequent accounts of increasing inequalities in resource access, the emergence of new conflicts and decreasing land health (Drew and Knutsson 2025), patterns consistent with a broader body of literature from the region (Rutten 1992; Fratkin and Roth 2006; Elliott 2016; Greiner 2017; Drew 2020). This is at least partly due to inadequate governance at state and sub-national levels, resulting in the weakening of customary resource management institutions and the loss of land under customary tenure, which, in turn, intensifies pressure on the remaining land and resources (Mwangi 2007). While the qualitative interviews indicate that this trend is more pronounced in West Pokot, it is nonetheless visible across all studied sites.

Furthermore, the annexation of East Africa’s drylands has been a feature of state intervention since the colonial administration. For example, by 1965, 94.6% of Uganda’s Karamoja sub-region had been designated for wildlife conservation (Rugadya and Kamusiime 2013). Since the turn of the century, there has been growing economic interest in the region as states and investors seek to exploit the mineral, energy-generating, and cultivable resources (Mosley and Watson 2016). While this commodification of land is an avenue for wealth accumulation for both state and non-state actors, the increasing focus on resource extraction in the region by state and private interests has challenged the sustainability of common livelihood practices among (agro-)pastoralists (Nakalembe et al. 2017; Lind et al. 2020a). Across the four studied sites, the decline in available land for livestock grazing and the concentration of livestock herds in smaller areas have overexploited and degraded land resources, including pasture, water and trees. While this linkage is widely recognised in the literature, its magnitude, variability across contexts, and interactions with other drivers of land degradation remain insufficiently quantified, particularly in relation to changes in mobility, land tenure, and governance arrangements. Our qualitative interviews include frequent narratives from individuals and families who, due to decreased access to fertile land for cultivation or livestock grazing, have become impoverished and even dropped out of pastoralism (Schumann et al. 2025).

Alongside commodification, weak legal protection of customary rights and ineffective administrative structures have enabled large-scale annexation of rangelands (Wily 2011; Ashukem 2020). Qualitative interviews with a diverse range of respondents across the four countries/districts studied reveal the high stakes involved in the governance of dryland resources and show that the resulting struggles and claims are reshaping resource distribution and conflict in the region (Drew and Knutsson 2025). More specifically, these interviews show how the combined processes of demarcation, formalisation, and increasing commodification of land are resulting in increasing conflict and tenure insecurity for the poorest and most vulnerable segment of the pastoralist population.

Although our analysis of quantitative and qualitative data shows that pastoralist communities across the Karamoja cluster face similar challenges regarding conflict and governance, use, and tenure related to dryland resources, it is important to also acknowledge that these challenges are highly complex and dynamic, with significant local and contextual differences across the region. Table 3 illustrates this by comparing land governance and conflict dynamics across the four districts and counties.

**Table 3:** Comparison of land and conflict governance dynamics across selected sites in the Karamoja cluster. Adapted from Drew and Knutsson (2025)

Land and conflict governance dynamics	Napak District, Uganda	Moroto District, Uganda	West Pokot County, Kenya	Turkana County, Kenya
Disarmament	Military intervention	Military intervention	No	Surrender of weapons campaigns
Land privatisation and commodification	Moderate	High	High	Low
Community Land Reform	Limited implementation	Limited implementation	Broad implementation	Broad implementation in the near future
Prevalence of land use conflicts	High	High	Moderate	Low

#### Livelihoods and food insecurity

Land degradation and subsequent reductions in the provision of ecosystem services lead to and are reinforced by unsustainable livelihood practices (e.g. tree cutting for charcoal burning and other uses, open mining, brewing), poor health, and conflict, although the relative strength and direction of these interactions vary across contexts and remain difficult to quantify empirically. These multi-fold challenges are exacerbated by poorly coordinated and disconnected development activities and governance interventions (IGAD 2019). Consequently, the Karamoja cluster has some of the lowest scores on human development indicators (Fig. 3). Climate change-related shocks, population pressure, conflicts, insecurity, and government interventions have pushed pastoralists to become increasingly sedentary, pursuing crop cultivation, agro-pastoralism, and urban wage labour (Nakalembe et al. 2017). A recent study conducted in the Karamoja cluster in Kenya and Uganda found that 22% of households have given up livestock keeping, primarily due to raids, security issues, and animal diseases, with higher rates observed in Uganda than in Kenya (Schumann et al. 2025).

These changes extend beyond livelihood strategies to social relations and gender norms, with important implications for food security, nutrition, and health. For example, the customary sharing of food and other assets within the community has decreased, leaving many households to fend for themselves (Barrantes and Caravani 2020). The decline in livestock herding has also made it difficult for many men to fulfil traditional provider roles, affecting gender dynamics and leading to family conflicts (FAO et al. 2023). Recent qualitative research from the Karamoja sub-region in Uganda further illustrates how livelihood changes intersect with gender roles, influencing well-being and household food security (Kyanjo et al. 2025). Cultural norms place primary responsibility for children’s nutrition and health on mothers, and social pressure to meet household financial needs has led many mothers to seek employment, balancing the benefits of increased income and autonomy with potential negative effects on child nutrition and health. Another qualitative study conducted in West Pokot identified both extrinsic and intrinsic barriers to food security (Kawira et al. 2025). Extrinsic barriers included environmental degradation and water-related challenges, policy-related challenges, non-ideal farming conditions, infrastructure constraints, and limited support from development partners. Intrinsic barriers to food security included cultural, economic, and social barriers. Three key cultural factors contributing to food insecurity were lack of support from men, lack of control of resources by women and heavy maternal workload.

The negative impact of environmental shocks on the livelihoods of the Karamoja people has partly been attributed to inadequate early-warning and early-response information systems regarding drought and insecurity, leaving communities vulnerable (IGAD 2019). In addition, poor infrastructure, including transport, and weak value chains and market linkages, are key challenges to livelihood improvement (IGAD 2019).

Food insecurity and malnutrition are major challenges in the Karamoja cluster (IPC 2024a; b). Almost 80% of households in the study by Schumann et al. (2025) were classified as severely food insecure based on the Household Food Insecurity Access Scale (HFIAS, Coates et al. 2007). These food-related challenges have considerable negative impacts on the region’s social and human development (Akiyode et al. 2017). Key underlying factors to acute food insecurity are land degradation, enduring state neglect, poverty, high staple food prices and price shocks, conflicts and insecurity, droughts, and floods, while key drivers for acute malnutrition are high levels of food insecurity, poor dietary intake and low dietary diversity, poor access to clean water and sanitation facilities, and diseases among humans, livestock, and crops (IPC 2024a; b). While these factors are widely recognised, their relative importance, interactions, and pathways to food insecurity and malnutrition vary across contexts and remain insufficiently disentangled empirically, underscoring the need for integrated and longitudinal analysis.

Low dietary diversity in the Karamoja cluster is prevalent and indicative of malnutrition and hidden hunger (i.e. lack of vitamins and minerals). Other indicators of undernutrition include low weight-for-height (wasting), indicating recent and severe weight loss, i.e. acute undernutrition, and low height-for-age (stunting), indicating chronic or recurrent undernutrition (WHO 2023). In both Uganda and Kenya, the prevalence of wasting, underweight, or stunting in the Karamoja cluster is higher than the national averages (Fig. 3; Supplementary Tables S1 and S2). Data from the Ugandan part of Karamoja show that among children aged 6–59 months, 12% were wasted, 34% were stunted, and 5% exhibited both conditions (Odei Obeng-Amoako et al. 2021). The risk of being both wasted and stunted was twice as high for children of underweight mothers, and stunting and wasting were associated with a higher incidence of acute respiratory infections and diarrhoea. Programmes for treating children with severe acute malnutrition have been implemented in the Karamoja cluster for decades (Mokori et al. 2023). However, common approaches to addressing malnutrition, such as reliance on food aid or the provision of single foods or nutrients, are widely regarded as unsustainable because they do not address underlying social–ecological drivers of food shortage. They might even worsen the situation by reinforcing dependence on external help, weakening traditional coping mechanisms, and contributing to the loss of knowledge of edible wild foods (Oduor et al. 2023, 2024).

An enduring challenge that influences and is influenced by many of the factors and challenges already mentioned is conflict (Onyango 2018), as discussed in Sect. “Inadequate governance: conflict, land reform and inequality”. The Karamoja cluster is no exception, as enduring conflict, especially around the increasing rush for land, continues to hamper the development of sustainable livelihood strategies (Abrahams 2021; Drew and Knutsson 2025). In the study by Schumann et al. (2025), 37% of households reported conflicts between communities, while 19% reported conflicts within their own community, with higher prevalences observed in Uganda compared to Kenya.

### Visioning the desired social–ecological system

Visioning constitutes a critical step in transformation processes because it enables diverse actors to co-create shared understandings of desirable sustainable futures and align collective action towards them (Moore and Milkoreit 2020). Our framework provides a generic vision of the desired social–ecological system, grounded in resilience and systems theory, and drawing on extensive experience from research and practice in drylands. To refine this vision and tailor it to the specific context of the Karamoja cluster, and to ensure it reflected the ambitions, priorities and perspectives of diverse actors, we conducted participatory scenario development workshops with various local stakeholder groups in Napak and Moroto districts and in Turkana and West Pokot counties.

Scenario development is a well-established method for collectively reconceptualising the future (Pereira et al. 2018). It provides an opportunity for participants to explore alternative desirable futures, the developments, actions, and political decisions that might lead there, and the uncertainties that could reshape the conditions necessary to enable the envisioned futures. Participatory scenario development and evaluation can facilitate the combination of actors’ different understandings of the challenges facing dryland social–ecological systems and enable them to work together to envision futures and transformative pathways that are novel yet realistic enough to inspire action. Based on their different positions, identities, stakes, and knowledges, actors will envision the challenges and solutions that lead to more desirable futures differently. The sustainability of transformations depends on privileging this diversity of positions, identities, stakes and knowledges (Jiren et al. 2023).

Building on the analysis of the current social–ecological system (section ‘Defining the challenges of the initial social–ecological system’), the workshops aimed to co-develop alternative visions of sustainable futures. Using our framework and the analysis of the current system as a common baseline and entry point, participants collectively discussed what desirable futures could look like and what system feedbacks could sustain them. Discussions were guided by the central question: What futures can we envision that amplify the feedback loop of “improved dryland health → enhanced ecosystem services → improved well-being and livelihoods → sustainable land management”? Participants were also asked to reflect specifically on how governance and social institutions could help strengthen this loop, and how such a system could, in turn, promote peace and equity.

Our summary of the challenges facing dryland ecosystems and communities in the Karamoja cluster highlights their interconnectedness, complexity and multidimensionality. Not surprisingly, different actors, including agro-pastoral and pastoralist communities, local administrators, county/district governments, private sector actors, NGOs, and academic scholars did not always agree on what they saw as the main drivers behind present social–ecological problems, the necessary conditions and actions envisioned to lead to more desirable futures, what at all constitutes such desirable futures, and at what scale challenges and their solutions can be most effectively addressed. Recognising and engaging with these multiple perspectives was central to the process of co-developing visions for sustainable transformation.

Two main desirable sustainable futures emerged during the envisioning workshops. They mirror both a synthesis of diverging arguments and perspectives expressed by different stakeholders during the workshops, and two contrasting views in a long-standing debate in the region and other dryland areas on whether enclosing communal land leads to negative social–ecological consequences by contributing to sedentarisation, commodification, and privatisation or if it, in contrast, can be used as a tool to restore degraded rangelands and enhance pasture and livestock productivity (Karmebäck et al. 2015; Nyberg et al. 2015; Knutsson et al. 2021). We named these two future scenarios “renewed mobile pastoralism” and “innovative agro-pastoralism”. Although both scenarios represent resilient and sustainable futures for the Karamoja cluster, they embody distinct configurations of social–ecological relationships. Table 4 summarises the two alternative scenarios and their relationships to the key elements of the current dryland social–ecological system.

**Table 4:** Summary of two alternative scenarios that emerged during the envisioning workshops: Renewed mobile pastoralism and Innovative agro-pastoralism

Scenario	Governance	Land tenure	Land use	Land health	Wellbeing		
					Health	Livelihoods	Peace
Renewed pastoralism	Centrally regulated mobility & grazing Negotiated agreements Multi-scale governance	Commons Community land (including informal subdivision) Negotiated access rights	Community-based resource use Grazing and water access corridors Herd diversification Silvopastoralism	Degradation control through multi-scale regulation Adaptive grazing Common water management	Market-based nutrition Dry season mobile health clinics Regional health programs	Climate resilience through mobility and herd diversity Food security through livelihood diversification Mobile schools	Enforcement of mobility and natural resource use regulations Inter-community political mobilisation
Innovative agro-pastoralism	Decentralised resource governance	Private land Community land Formal access rights	Household-based agricultural intensification, including fodder production	Degradation control through local management Local/private water management	Nutrition through intensive, self-sufficient & diverse food production Urban centre health clinics Local health programs	Intensified food production Climate resilience through crop & livestock diversification Sedentary schools	Clear and formalised boundaries Household-resource control

Renewed mobile pastoralism builds on the revitalisation of traditional social–ecological systems that enable and support the seasonal mobility of people and livestock, ensuring inclusive community access to grazing and water resources. In this vision, food security and sustainable livelihoods are ensured by regulation of mobility and resource use, coordinated through negotiated agreements among communities and authorities. The vision is underpinned by multi-scale and collaborative governance, linking local institutions to regional and national authorities and their economic, environmental and political plans and priorities, to harmonise resource use across administrative boundaries. Land remains largely under communal tenure, functioning as commons or community land with informal subdivisions and maintained through negotiated access rights that ensure inclusivity and flexibility. Resource use follows community-based management, supported by grazing and water access corridors that maintain ecological connectivity between seasonal pastures and water points. Dryland health is safeguarded through adaptive grazing management practices, common water management, and multi-scale regulations designed to sustain ecosystem services. Well-being is supported through market-based nutrition, mobile health clinics, and regional health programmes, complemented by mobile schools that ensure educational access for transhumant families. Livelihood resilience is derived from herd diversity, mobility, and livelihood diversification, whereas peace and stable relations between communities depend on regulating mobility and resource use and on inter-community political mobilisation and collaboration centred around common pastoralist identities, social–ecological challenges, and livelihood needs and priorities.

By contrast, innovative agro-pastoralism entails a more sedentary and decentralised system of resource governance in which pastoral and agricultural practices are integrated at the household and community levels. Land tenure is mixed, combining private and community land tenure with formalised access rights to reduce disputes and clarify responsibilities. Land use focuses on household-based agricultural intensification, including fodder production to sustain livestock productivity. Dryland health is maintained through local rangeland management and private or communal water management, ensuring resource efficiency and stability at the local scale. Human well-being is enhanced through intensive, self-sufficient, and diversified food production, supported by local health programmes, urban-centre health clinics, and sedentary schooling that strengthens links between families and services. Livelihood resilience arises from crop and livestock diversification, while peace and stability are supported by clear and formalised land boundaries and stronger household-level resource control.

The two scenarios were evaluated through a participatory approach in which participating community members, along with relevant local government and civil society representatives, evaluated them according to two complementary aspects. First, by assigning scores to each scenario in relation to a limited number of criteria. These criteria were formulated based on key feedbacks between dryland health, ecosystem functioning and provision of ecosystem services, and livelihoods & human well-being. Second, by assigning relative weights to each of the evaluation criteria to indicate the importance they attributed to each one. The evaluation provided a transparent account not only of which scenario was considered more desirable by different groups but also why they did so.

Although renewed mobile pastoralism and innovative agro-pastoralism differ in their institutional and governance arrangements and degrees of mobility, both alternative envisioned futures strengthen key feedbacks between dryland health, ecosystem functioning and the provision of ecosystem services, and livelihoods and human well-being. In practice, elements of these two visions may coexist or be combined at the landscape level. The following section explores the pathways and mechanisms through which progress towards these envisioned futures could be realised.

### Identifying and operationalising pathways to change

The scenario development workshops were also used to discuss potential pathways towards desirable sustainable futures. While the values, perspectives and positions of different stakeholders and actors can make it challenging to agree upon common pathways, they simultaneously provide the basis for the diversity of knowledges that just and sustainable transformations depend upon (IPBES 2024b). Collaboration among stakeholders with diverse knowledges is required to explore possible synergies and trade-offs between various pathways. As previously discussed, all three lenses of sustainable transformation (i.e. *structural, systemic, and enabling/grassroots*) are needed to understand and operationalise potential pathways.

We illustrate this with two key, potentially complementary, human-driven pathways towards sustainability transformations of dryland social–ecological systems: (1) the restoration of degraded drylands and (2) functional and inclusive dryland governance. These general pathways are relevant to both envisioned futures—renewed mobile pastoralism and innovative agro-pastoralism—although more detailed and context-specific strategies will be needed to operationalise each vision. These examples also demonstrate how the three lenses of our framework provide complementary insights for designing actionable strategies and guiding transformative change.

#### Transforming livelihoods and landscapes through the restoration of degraded drylands

A healthy biosphere is a prerequisite for sustainable development and human well-being (Folke et al. 2016). Halting land degradation and restoring degraded lands are key transformative pathways directed at ensuring healthy land ecosystems, thereby improving their capacity to deliver ecosystem services that are vital for people’s livelihoods, health and well-being (IPBES 2018). Restoration is central to addressing the three-fold crises of climate change, land degradation, and biodiversity loss and achieving transformative change. Ecosystem restoration is high on the policy agenda, as evidenced by global initiatives such as the UN Decade on Ecosystem Restoration (2020–2030) and the Bonn Challenge. In the drylands of East Africa, there are several regional and national restoration initiatives, including the African Forest Landscape Restoration Initiative (AFR100) and related national efforts (Pasiecznik and Reij 2020). For example, Kenya and Uganda have committed to restoring 5.1 and 2.5 Mha, respectively, under AFR100. The majority of this will take place in drylands.

Maes et al. (2024) argue that, given the complex nature of ecosystems, incorporating complex systems thinking into ecosystem restoration could significantly improve restoration outcomes. By considering nonlinearity, regime shifts, ecological resilience, and ecological feedbacks when planning their interventions, restoration practitioners can influence degradation and recovery trajectories, thus impacting restoration outcomes (Maes et al. 2024). In some parts of the Karamoja cluster, severe soil erosion in the form of gully erosion is a widespread land degradation issue (Churu et al. 2024; Bargués-Tobella et al. 2025). Positive feedback loops reinforce the degradation process; bare and degraded soil results in higher runoff rates, which, in turn, lead to reduced soil moisture available to plants and increased erosion, restricting the recovery of vegetation and soil health. Restoration interventions should aim to disrupt this degradation cycle and the feedbacks that drive it to speed up recovery (Bargués-Tobella et al. 2024). This often requires multiple complementary interventions and technologies such as an initial reduction of grazing intensity (e.g. grazing exclosures), structural barriers (e.g. bunds, check dams) that retard surface runoff and retain eroded sediment and soil moisture, or active reseeding of perennial grasses and other permanent vegetation (Liniger and Critchley 2007). Recent work in the Karamoja cluster shows that combining grazing exclosures with active reseeding of perennial grasses and legumes, together with manure application, can substantially increase herbaceous biomass and cover, as well as plant community structure, underscoring the value of integrated restoration technologies (Nyaga et al. 2025). Examples of gully rehabilitation technologies that have proven successful in the drylands of East Africa include deep trenches, gully-wall reshaping, check dams (loose stone, or gabions), and vegetative stabilisation (Mureithi et al. 2025).

Given the high prevalence of undernutrition and the persistent challenge of food insecurity and low dietary diversity in the Karamoja cluster (IPC 2024a; b), nutrition-sensitive restoration interventions that support sustainable food systems and healthier and diverse diets are critical. Examples of such interventions include the promotion of a diversity of plants, including trees and crops, that provide fruits, nuts, roots, tubers and leafy vegetables that constitute key sources of macro- and micronutrients and contribute to dietary quality, thus promoting both dietary and plant diversity (Oduor et al. 2023, 2024, 2025). The planning and prioritisation of restoration interventions must consider key decision criteria such as ecological thresholds, labour and cost constraints, tenure security and resource use rights, and gendered labour dynamics, including women’s time and workload (Pasiecznik and Reij 2020; Maes et al. 2024).

The Karamoja cluster encompasses different but interdependent land uses and management practices—including rangelands, croplands, or conservation areas—under various governance systems. It is thus essential to use a landscape approach to restoration, ensuring that different priorities can be balanced and optimised and promoting landscape connectivity, which is a vital element for pastoralist livelihoods and the conservation of wildlife and biodiversity at large (Crego et al. 2021). Other examples of systemic change are policies that promote the restoration of degraded drylands, as well as institutional and regulatory reforms that create enabling conditions for restoration initiatives.

In addition to systemic transformations, attaining more sustainable and equitable restoration requires transformations of the underlying structures of existing political, economic and social relations in society at multiple scales that underpin unsustainable land use and management practices and unequal and unfair control over natural resources (Osborne et al. 2021; Elias et al. 2022; Löfqvist et al. 2022). For instance, institutions governing resource rights and access at various scales may require shifts in structure, power and control for system changes in human–environment relations to be sustainable. Examples of such structural change could be, as we will return to in more detail in section ‘Transforming livelihoods and landscapes through functional and inclusive dryland governance’, the reform of resource access and tenure rights of marginalised groups, including women, that would ensure an equitable distribution of costs and benefits from restoration, or fair compensation to pastoralist communities for loss of common property natural resources in connection to state- or private investments.

Community-led restoration efforts can be highly successful (Pasiecznik and Reij 2020), while top-down approaches often overlook the human and social dimensions of ecosystem restoration (Elias et al. 2022). Facilitating grassroots restoration is critical to achieving both ecological and social restoration goals (as defined by different actors) at scale (Elias et al. 2022). Enabling approaches to transformation may include creating the conditions for political mobilisation at the community level to ensure that individual and collective rights to natural resources are respected, strengthening the capacity of farmer/pastoralist and other producer associations, strengthening grassroots institutions such as loan associations, promoting farmer-to-farmer restoration training and knowledge exchange, supporting the collective agency of local actors in restoration planning and implementation, and initiatives by state and non-state actors to empower women to initiate restoration, e.g. by establishing kitchen gardens to enhance food security for children. Furthermore, a key to ensuring the effectiveness of land policies and reforms aimed at structural or systemic change for land restoration is the identification of context-sensitive incentives for restoration, as well as disincentives for land degradation, that enable the willingness and capacity of communities and individuals to partake in restoration (Wainaina et al. 2021). Examples of such incentives include Payments for Ecosystem Services (PES), management rights, subsidies, technical assistance, carbon financing projects, prizes for lead farmers and pastoralists, tax incentives, local by-laws supporting sustainable grazing management, and negotiated grazing agreements that balance restoration and pastoralist needs (Pasiecznik and Reij 2020; Moon et al. 2021; Wainaina et al. 2021; Yan et al. 2022; Jain et al. 2025). Disincentives for unsustainable land management may include restrictions or penalties on unsustainable practices such as tree cutting or overgrazing. Organisations responsible for the implementation of these incentives and disincentives vary depending on the mechanism and can include actors from the public, private and third sectors.

#### Transforming livelihoods and landscapes through functional and inclusive dryland governance

As the section ‘Inadequate governance: conflict, land reform and inequality’ shows, pastoralist communities in East Africa, in general, and the Karamoja cluster in particular, suffer from inadequate governance, which has enabled ongoing acquisition and annexation of pastoralist land by the state and private investors, and led to the erosion of rural communities’ rights over, and capacity to access and govern resources (Wily 2011; Onyango 2018; Ashukem 2020; Flintan et al. 2021). The need for more functional and inclusive resource governance has become especially acute over the last decade, as the East African drylands have become the focal point for increasing state and private land appropriation to enable mining and energy exploitation, large-scale infrastructure development and agricultural expansion (Lind et al. 2020b; Aalders et al. 2021), triggering new inter- and intra-community conflict dynamics and putting the access and control over vital land-based resources at risk for the poorest and most vulnerable section of the pastoralist population.

A transformation that ensures not only the legal protection of pastoralist communities’ right to land but also an equitable distribution of land-based resources, as well as flexible, negotiated seasonal access to resources based on mobility, requires a delicate combination of *structural*, *systemic* and *enabling/grassroots* change. While community land reforms introduced in both Uganda and Kenya potentially constitute a structural change in how drylands are governed, as they open for more secure and formally recognised collective land rights, they have not yet delivered the necessary governance transformation. Our extensive, qualitative research on land governance on the Uganda–Kenya border confirms the conclusion offered by Robinson and Flintan (2022) that the introduced legal frameworks must be complemented by legal provisions that not only provide security against alienation and fragmentation of land but also legitimise collective management and allow for flexible natural resource access and management. However, formal legal provisions that are sensitive to pastoralist livelihoods and knowledges do not by themselves lead to equitable resource distribution and sustainable land use. The pathway towards functional and inclusive dryland governance requires coordination and reform at the landscape level, grounded in a thorough understanding of the complex dynamics of the drylands as a social–ecological system. This implies the capacity to balance the right to sustainable livelihoods among pastoralist communities, the potential of drylands to deliver valuable energy and mineral resources for a growing global green economy, and the need for dryland restoration and conservation. Such a balancing act rests on acknowledging the complex feedback loops that characterise dryland system dynamics, as well as actively promoting the inclusion and participation of pastoralist communities, individuals, and organisations in the governance, management and use of dryland resources. In particular, forceful and effective political and legal measures as part of a more structural change by states are needed to ensure the protection of individual and community resource rights amid increasing national and international economic interests in the use and exploitation of dryland resources. Inspiration for such measures can for example be drawn from successful implementation of mechanisms for community consultation as well as principles of free, prior, and informed consent in negotiations of dryland natural resource investments (Szoke-Burke and Cordes 2020), but also from legal proceedings where courts of law have ruled in favour of pastoralist communities who have had their resource rights neglected by private and state actors in connection to large-scale investments (Mósesdóttir 2024; Bülow and Nweke-Eze 2025). In line with this quality of the transformation pathway, the capacity of state and non-state actors to implement reforms, legislation and policies to enhance equitable resource rights and access for pastoralist individuals and communities needs to be significantly strengthened. The transformation pathway we outline here furthermore stands in stark contrast to the ongoing economic and political marginalisation of pastoralist communities, including the devaluation and stigmatisation of pastoralism as a way of life. As such, our proposed transformation pathway draws hopeful inspiration from both the enabling/grassroots change that current trends of new forms of pastoralist political mobilisation in different parts of the world constitute (Upton , 2014), and the more system-oriented change that the IYRP 2026 aims for by raising awareness about and advocating for the value of pastoralist rights, knowledge and practices for sustainable rangeland governance and management.

#### Considering trade-offs and synergies among transformation pathways

For multiple transformation pathways to concertedly lead to a resilient social–ecological system, the often complex and dynamic trade-offs and synergies among parallel, and sometimes competing, pathways and potential desirable futures must be carefully analysed and inclusively engaged within a multi-sectoral and multi-stakeholder setting. Although it is beyond the scope of this paper to provide a detailed analysis of trade-offs and synergies between the two exemplary pathways presented, a few principal observations can be made. First, there is substantial empirical evidence, particularly from the Kenyan drylands, of problematic consequences of land restoration and conservation initiatives for pastoralist control over and access to land-based resources, which, in turn, put the resilience of this mode of conservation at risk. For example, Pas et al. (2023) show how the spread of community-based conservancies across dryland Kenya during the last 30 years has fundamentally reorganised land governance and resulted in hardened conflicts between wildlife conservation and pastoralist communities, as well as between and within pastoralist communities. On the other hand, the development of new approaches and forms of land restoration and conservation that are guided by resilience principles and that connect an increasingly economically and politically powerful international land restoration and conservation agenda with increased local community empowerment, influence over, and active participation in restoration, can potentially open the door for turning existing trade-offs between restoration of degraded lands and good governance into synergies (Sigman and Elias 2021). Furthermore, sustainability transformations and resilient social–ecological systems come about through the interplay between processes at different scales and levels of society (Geels 2019; Folke et al. 2020). Both restoration and good governance of dryland social–ecological systems require effective and transparent processes of change at and across different scales.

## Concluding discussion

### Lessons learned from applying the framework in the context of the Karamoja cluster

The framework we propose provides a structured approach to promoting, preparing for and guiding sustainability transformations in drylands, offering an entry point for engagement across different disciplines and sectors. Its relative simplicity is a strength: it enables different stakeholder groups, including local communities, local and national authorities, researchers, development practitioners, and private sector actors, to grasp key concepts and foster shared understanding. This accessibility further facilitates collective action and promotes cross-sectoral dialogue. At the same time, this simplicity may be perceived as a limitation by specialists accustomed to highly nuanced analyses. The framework should therefore be seen as a starting point, from which more detailed investigation and planning can follow. Our experience demonstrates that this simplicity and accessibility encourage transdisciplinary collaboration that might not emerge with more complex frameworks.

The proposed framework stresses the importance of creating enabling conditions for local agency if sustainable transformations are to be realised. In practice, this is one of the most difficult dimensions of transformation to be achieved. As illustrated by the application of the framework in the Karamoja cluster, powerful actors such as governments, investors, and local elites tend to limit the room for pastoralist individuals and communities to influence strategic political decisions and meaningfully engage in transformative change and access its benefits. An important lesson learned in this regard is that the scope for transformative change from below is highly dependent on parallel changes in the structural and systemic conditions that shape the extent to which local agency is allowed to influence processes of social–ecological change (Ndiritu et al. 2025).

Another key limitation, shared by any future-oriented framework, is that the envisioned futures, solutions, and pathways developed from it tend to be drawn from present and past trajectories and experiences. As illustrated in section ‘Applying the framework: The case of the Karamoja cluster in the drylands of East Africa’, our application of the framework using the Karamoja cluster case reflects several years of work within a research project. While we have implemented the steps within the “preparing for transformation” phase, this remains an iterative process, and the examples presented are thus limited to what has been achieved to date.

Furthermore, scenarios, pathways, or envisioned futures used to guide transformative change primarily constitute synthesised and idealised points of departure that, by necessity, will unfold differently and lead to different outcomes across diverse local contexts. Such variation reflects differences in environmental endowment, ethnic composition, political dynamics, governance structures, etc. The experience of working with alternative scenarios and pathways across four sites within the *Drylands Transform* project underscores the importance of ensuring room for context-specific influence in their design and evaluation.

Although the framework may appear linear, it is designed to support cycles of reflection, learning, and adaptation, consistent with the importance placed on such iterative cycles in the literature on change management, adaptive management and theory of change (Williams 2011; Stein and Valters 2012; Walker 2017). It is applicable even in early stages of system characterisation, helping stakeholders identify key challenges, envision desired sustainable futures, and plan interventions while simultaneously considering the data and monitoring required to inform iterative decision-making. To support ongoing learning, we recommend establishing targeted monitoring systems that include both ecological and social indicators. The LDSF (Vågen and Winowiecki 2023), for example, provides a structured approach for assessing and tracking multiple indicators of land health at the landscape scale and can serve as a useful model for similar initiatives. However, because frameworks such as the LDSF focus on specific components of the social–ecological system, they should be carefully combined with monitoring of other system dimensions to allow meaningful analyses of interactions.

Our empirical work, coupled with participatory scenario development workshops, highlighted the importance of integrating multiple knowledge systems, including local, indigenous, and scientific knowledge. This integration enhances understanding of key feedbacks, trade-offs, and synergies in social–ecological systems and strengthens the legitimacy and sustainability of transformations. Our experience also suggests that, to increase the potential of the developed transformation pathways to promote real transformative change, key stakeholders with interests, responsibilities, and agency in relation to dryland social–ecological change should be engaged throughout all three steps in preparing for transformation. Dedicated participation by key community-based and civil society organisations, private-sector networks, regional collaborative platforms, and local and national government representatives increases the willingness and openness of important agents of change to take part in transformative collective action.

### Transferability of the framework to other dryland contexts

Our work in the Karamoja cluster, a cross-border region with substantial social–ecological variability, demonstrates that the conceptual framework is flexible and applicable across diverse dryland contexts. The main elements of the framework—the general structure of the three key phases of transformation and associated actions, and the lenses of transformation (*systemic, structural, enabling/grassroots*)—are broadly generalisable. At the same time, transferring the framework beyond Karamoja rests on several assumptions and boundary conditions. First, the framework assumes that users can co-define (at least in a minimal way) the key social–ecological challenges, system feedbacks, and governance dynamics relevant to the focal dryland social–ecological system, drawing on a combination of available evidence and stakeholder knowledge. Second, it assumes sufficient scope for inclusive engagement across stakeholder groups to support shared problem framing and the co-development of desired futures and pathways, recognising that such engagement might be constrained by power asymmetries and conflict dynamics. Third, the framework must be adapted to local social–ecological contexts, including political and institutional realities (particularly the specific configurations of land tenure and access regimes, mobility and resource governance arrangements, and the main sources of livelihood insecurity and inequality), since these conditions shape both feasible pathways and likely trade-offs. When applied with these adaptations and with explicit attention to data constraints and uncertainty, the framework offers a valuable tool to help practitioners better prepare for and support transformations towards sustainability in drylands.

## Conclusions

This paper presents a conceptual framework to support social–ecological transformations towards sustainability in dryland systems, integrating systemic, structural and enabling/grassroots lenses along three key phases of transformation. By illustrating the application of the framework through the Karamoja cluster case, we show how it can be used to diagnose interlinked challenges, support participatory visioning of desired social–ecological futures, and identify context-specific pathways to change. The framework is intended as a flexible and reflexive tool rather than a prescriptive blueprint, helping researchers, practitioners and decision-makers engage more explicitly with system dynamics and feedbacks, structural constraints and conditions for local agency when preparing for sustainability transformations. When applied with attention to local social–ecological contexts, the framework can contribute to more coherent, inclusive and durable transformation processes in drylands.

## Supplementary Information

Below is the link to the electronic supplementary material.

Supplementary file1 (PDF 517 KB)

## Data Availability

The data and R code used to produce Fig. 3 are available in a figshare repository (https://doi.org/10.6084/m9.figshare.30380197).

## References

[CR1] Aalders, J.T., J. Bachmann, P. Knutsson, and B. Musembi Kilaka. 2021. The making and unmaking of a megaproject: Contesting temporalities along the LAPSSET corridor in Kenya. *Antipode* 53: 1273–1293. 10.1111/anti.12720.

[CR2] Abrahams, D. 2021. Land is now the biggest gun: Climate change and conflict in Karamoja, Uganda. *Climate and Development* 13: 748–760. 10.1080/17565529.2020.1862740.

[CR3] Adeel, Z., U. Safriel, D. Niemeijer, and R. White. 2005. *Ecosystems and human well-being: Desertification synthesis*. Washington: World Resources Institute (WRI).

[CR4] Akall, G. 2021. Effects of development interventions on pastoral livelihoods in Turkana County, Kenya. *Pastoralism* 11: 23. 10.1186/s13570-021-00197-2. PMC8610612 34840712

[CR5] Akiyode, O.O., A. Tumushabe, H. Katongole, and P. Onu. 2017. Climate change, food security and environmental security: A conflict inclination assessment of Karamoja region of Uganda. *International Journal of Scientific World* 5: 172–176. 10.14419/ijsw.v5i2.8458.

[CR6] Allred, B.W., S.E. McCord, T.J. Assal, B.T. Bestelmeyer, C.S. Boyd, A.C. Brooks, S.M. Cady, M.C. Duniway, et al. 2025. Sentinel-2 based estimates of rangeland fractional cover and canopy gap class for the western United States. *Scientific Data* 12: 1889. 10.1038/s41597-025-06160-9. PMC12663396 41315284

[CR7] Arias, P.A., N. Bellouin, E. Coppola, R.G. Jones, G. Krinner, J. Marotzke, V. Naik, M.D. Palmer, et al. 2021. Technical summary. In *Climate change 2021: The physical science basis. Contribution of working group I to the sixth assessment report of the intergovernmental panel on climate change*, ed. V. Masson-Delmotte, P. Zhai, A. Pirani, S.L. Connors, C. Péan, S. Berger, N. Caud, Y. Chen, et al., 33–144. Cambridge: Cambridge University Press.

[CR8] Ashukem, J.-C.N. 2020. Land grabbing and customary land rights in Uganda: A critical reflection of the constitutional and legislative right to land. *International Journal on Minority and Group Rights* 27: 121–147. 10.1163/15718115-02701003.

[CR9] Ayana, E.K., P. Ceccato, J.R.B. Fisher, and R. DeFries. 2016. Examining the relationship between environmental factors and conflict in pastoralist areas of East Africa. *Science of the Total Environment* 557–558: 601–611. 10.1016/j.scitotenv.2016.03.102. 27037881

[CR10] Bargués-Tobella, A., L.A. Winowiecki, D. Sheil, and T.G. Vågen. 2024. Determinants of field-saturated soil hydraulic conductivity across Sub-Saharan Africa: Texture and beyond. *Water Resources Research* 60: e2023WR035510. 10.1029/2023WR035510.

[CR11] Bargués-Tobella, A., L.A. Winowiecki, M.N. Ahmad, W. Kiunga, M. Lundberg Ingemarsson, A. Mohamoud, D. Mpairwe, D. Munyua, et al. 2025. Scaling rangeland restoration in East Africa through synergies in the biodiversity-waterclimate nexus. In *XII international rangeland congress, Adelaide, Australia*, 577–580.

[CR12] Barrantes, A., and M. Caravani. 2020. Situational analysis of food, nutrition and income security in Karamoja: A normalizing view of Karamoja. World Food Programme. https://www.wfp.org/publications/situation-analysis-food-nutrition-and-income-insecurity-karamoja-2020. Accessed 10 October 2025.

[CR13] Bennett, E.M., R. Biggs, G.D. Peterson, and L.J. Gordon. 2021. Patchwork Earth: Navigating pathways to just, thriving, and sustainable futures. *One Earth* 4: 172–176. 10.1016/j.oneear.2021.01.004.

[CR14] Biggs, O., M. Schülter, and M.L. Schoon. 2015. *Principles for building resilience: Sustaining ecosystem services in social-ecological systems*. Cambridge: Cambridge University Press.

[CR15] Biggs, R., A. De Vos, R. Preiser, H. Clements, K. Maciejewski, and M. Schlüter. 2021. *The Routledge handbook of research methods for social-ecological systems*. London: Taylor & Francis.

[CR16] Blake, W.H., A. Rabinovich, M. Wynants, C. Kelly, M. Nasseri, I. Ngondya, A. Patrick, K. Mtei, et al. 2018. Soil erosion in East Africa: An interdisciplinary approach to realising pastoral land management change. *Environmental Research Letters* 13: 124014. 10.1088/1748-9326/aaea8b.

[CR17] Bülow, N., and C. Nweke-Eze. 2025. Investigating green colonialism and energy justice in the development of Lake Turkana wind power project in Kenya. In *The emerald handbook of sustainable energy transition and social justice: Contemporary issues and debates in the Global South*, ed. J.O. Irene, B.N. Irene, K.O. Omeihe, and R. Frank. Bingley: Emerald Publishing Limited.

[CR18] Caniglia, B.S., and B. Mayer. 2021. Socio-ecological systems. In *Handbook of environmental sociology*, ed. B.S. Caniglia, A. Jorgenson, S.A. Malin, L. Peek, D.N. Pellow, and X. Huang, 517–536. Cham: Springer International Publishing.

[CR19] Caravani, M. 2024. The failing aid complex in Uganda’s Northeast. *The European Journal of Development Research* 36: 1573–1592. 10.1057/s41287-024-00651-y.

[CR20] Carrier, N., and H.H. Kochore. 2014. Navigating ethnicity and electoral politics in northern Kenya: The case of the 2013 election. *Journal of Eastern African Studies* 8: 135–152. 10.1080/17531055.2013.871181.

[CR21] Cederlöf, G., and A. Loftus. 2023. *Discovering political ecology*. London: Routledge.

[CR22] Cherlet, M., C. Hutchinson, J. Reynolds, J. Hill, S. Sommer, and G. Von Maltitz. 2018. *World atlas of desertification: Rethinking land degradation and sustainable land management*. Luxembourg: Publications Office of the European Union. https://publications.jrc.ec.europa.eu/repository/handle/JRC111155. Accessed 10 October 2025.

[CR23] Churu, H., S. Kamau, W. Ng’etich, K. Magiroi, B. Alkamoi, S. Kebeney, F. Wamalwa, and J. Mumo. 2024. Drivers of gully erosion and its socio-economic and environmental effects in a tropical semi-arid environment. *Asian Journal of Soil Science and Plant Nutrition* 10: 13–26. 10.9734/ajsspn/2024/v10i2256.

[CR24] Coates, J., A. Swindale, and P. Bilinsky. 2007. Household food insecurity access scale (HFIAS) for measurement of food access: Indicator guide—Version 3. Washington: FHI 360/FANTA. https://www.fao.org/fileadmin/user_upload/eufao-fsi4dm/doc-training/hfias.pdf. Accessed 10 October 2025.

[CR25] Crego, R.D., H.B.M. Wells, K.S. Ndung’u, L. Evans, R.N. Nduguta, M.A. Chege, M.B. Brown, J.O. Ogutu, et al. 2021. Moving through the mosaic: Identifying critical linkage zones for large herbivores across a multiple-use African landscape. *Landscape Ecology* 36: 1325–1340. 10.1007/s10980-021-01232-8.

[CR26] Czuba, M.K. 2019. Projection of state power: State-making in the dryland periphery of Ethiopia, Kenya, and Uganda. PhD thesis. Toronto: Department of Political Science, University of Toronto.

[CR27] de Haan, C. 2016. Prospects for livestock-based livelihoods in Africa’s drylands. Washington: World Bank Group. http://documents.worldbank.org/curated/en/485591478523698174/Prospects-for-livestock-based-livelihoods-in-Africa-s-drylands. Accessed 10 October 2025.

[CR28] Davies, J., L. Poulsen, B. Schulte-Herbrüggen, K. Mackinnon, N. Crawhall, W.D. Henwood, N. Dudley, J. Smith, et al. 2012. *Conserving dryland biodiversity*. Nairobi, Bonn: IUCN, UNEP-WCMC, UNCCD. https://iucn.org/resources/publication/conserving-dryland-biodiversity. Accessed 10 October 2025.

[CR29] Díaz, S., S. Demissew, J. Carabias, C. Joly, M. Lonsdale, N. Ash, A. Larigauderie, J.R. Adhikari, et al. 2015. The IPBES conceptual framework—Connecting nature and people. *Current Opinion in Environmental Sustainability* 14: 1–16. 10.1016/j.cosust.2014.11.002.

[CR30] Drew, J. 2020. Meanings of place & struggles for inclusion in the Lake Turkana wind power project. In *Land investment and politics: Reconfiguring Eastern Africa’s pastoral drylands*, ed. J. Lind, D. Okwena, and I. Scoones, 66–77. Woodbridge: Boydell & Brewer.

[CR31] Drew, J., and P. Knutsson. 2025. Boundary-making, tenure insecurity, and conflict: Regional dynamics of land tenure change and commodification in East Africa’s pastoralist rangelands. *World Development* 194: 107068. 10.1016/j.worlddev.2025.107068.

[CR32] Easdale, M.H., and S.E. Domptail. 2014. Fate can be changed! Arid rangelands in a globalizing world—A complementary co-evolutionary perspective on the current ‘desert syndrome.’ *Journal of Arid Environments* 100–101: 52–62. 10.1016/j.jaridenv.2013.10.009.

[CR33] Elias, M., M. Kandel, S. Mansourian, R. Meinzen-Dick, M. Crossland, D. Joshi, J. Kariuki, L.C. Lee, et al. 2022. Ten people-centered rules for socially sustainable ecosystem restoration. *Restoration Ecology* 30: e13574. 10.1111/rec.13574.

[CR34] Elliott, H. 2016. Planning, property and plots at the gateway to Kenya’s ‘new frontier.’ *Journal of Eastern African Studies* 10: 511–529. 10.1080/17531055.2016.1266196.

[CR35] Enns, C. 2019. Infrastructure projects and rural politics in northern Kenya: The use of divergent expertise to negotiate the terms of land deals for transport infrastructure. *The Journal of Peasant Studies* 46: 358–376. 10.1080/03066150.2017.1377185.

[CR36] FAO, IFAD, UNICEF, WFP, and WHO. 2021. The state of food security and nutrition in the world 2021: Transforming food systems for food security, improved nutrition and affordable healthy diets for all. Rome: FAO. https://www.fao.org/3/cb4474en/cb4474en.pdf. Accessed 10 October 2025.

[CR37] FAO, IGAD, and Interpeace. 2023. Conflict, climate change, food security, and mobility in the Karamoja Cluster—A study to analyse interactions among conflict, food security, climate change, migration and displacement factors. Rome: FAO. https://openknowledge.fao.org/handle/20.500.14283/cc7672en. Accessed 10 October 2025.

[CR38] Feng, S., and Q. Fu. 2013. Expansion of global drylands under a warming climate. *Atmospheric Chemistry and Physics* 13: 10081–10094. 10.5194/acp-13-10081-2013.

[CR39] Flintan, F.E., L.W. Robinson, and M. Allen. 2021. A review of tenure and governance in the pastoral lands of East and West Africa. SPARC: Supporting Pastoralism and Agriculture in Recurrent and Protracted Crises. https://www.sparc-knowledge.org/publications-resources/review-tenure-and-governance-pastoral-lands-east-and-west-africa. Accessed 10 October 2025.

[CR40] Folke, C., S.R. Carpenter, B. Walker, M. Scheffer, T. Chapin, and J. Rockström. 2010. Resilience thinking: Integrating resilience, adaptability and transformability. *Ecology and Society* 15: 20.

[CR41] Folke, C., Å. Jansson, J. Rockström, P. Olsson, S.R. Carpenter, F.S. Chapin, A.-S. Crépin, G. Daily, et al. 2011. Reconnecting to the Biosphere. *Ambio* 40: 719. 10.1007/s13280-011-0184-y. PMC3357749 22338712

[CR42] Folke, C., R. Biggs, A.V. Norström, B. Reyers, and J. Rockström. 2016. Social-ecological resilience and biosphere-based sustainability science. *Ecology and Society* 21: 41. 10.5751/ES-08748-210341.

[CR43] Folke, C., H. Österblom, J.-B. Jouffray, E.F. Lambin, W.N. Adger, M. Scheffer, B.I. Crona, M. Nyström, et al. 2020. An invitation for more research on transnational corporations and the biosphere. *Nature Ecology & Evolution* 4: 494–494. 10.1038/s41559-020-1145-2. 32112049

[CR44] Fratkin, E., and E.A. Roth. 2006. *As pastoralists settle: Social, health, and economic consequences of the pastoral sedentarization in Marsabit District, Kenya*. New York: Springer.

[CR45] Fukui, K., and J. Markakis. 1994. Introduction. In *Ethnicity and conflict in the Horn of Africa*, ed. K. Fukui and J. Markakis. London: James Currey.

[CR46] Geels, F.W. 2018. Socio-technical transitions to sustainability. *Oxford Research Encyclopedia of Environmental Science*. 10.1093/acrefore/9780199389414.013.587.

[CR47] Geels, F.W. 2019. Socio-technical transitions to sustainability: A review of criticisms and elaborations of the multi-level perspective. *Current Opinion in Environmental Sustainability* 39: 187–201. 10.1016/j.cosust.2019.06.009.

[CR48] Gibbs, H.K., and J.M. Salmon. 2015. Mapping the world’s degraded lands. *Applied Geography* 57: 12–21. 10.1016/j.apgeog.2014.11.024.

[CR49] Green, E.D. 2008. Decentralisation and conflict in Uganda. *Conflict, Security & Development* 8: 427–450. 10.1080/14678800802539317.

[CR50] Greiner, C. 2013. Guns, land, and votes: Cattle rustling and the politics of boundary (re) making in Northern Kenya. *African Affairs* 112: 216–237. 10.1093/afraf/adt003.

[CR51] Greiner, C. 2017. Pastoralism and land-tenure change in Kenya: The failure of customary institutions. *Development and Change* 48: 78–97. 10.1111/dech.12284.

[CR52] Haines, A., R.S. Kovats, D. Campbell-Lendrum, and C. Corvalan. 2006. Climate change and human health: Impacts, vulnerability and public health. *Public Health* 120: 585–596. 10.1016/j.puhe.2006.01.002. 16542689

[CR53] Holling, C.S. 1973. Resilience and stability of ecological systems. *Annual Review of Ecology and Systematics* 4: 1–23.

[CR54] Huang, J., Y. Li, C. Fu, F. Chen, Q. Fu, A. Dai, M. Shinoda, Z. Ma, et al. 2017. Dryland climate change: Recent progress and challenges. *Reviews of Geophysics* 55: 719–778. 10.1002/2016rg000550.

[CR55] IGAD. 2019. Mapping of actors in Karamoja cluster. https://resilience.igad.int/wp-content/uploads/2022/05/Mapping-of-Actors-in-Karamoja-Cluster-2019.pdf. Accessed 10 October 2025.

[CR56] IGAD. 2022. IGAD cluster 1 (Karamoja cluster). https://resilience.igad.int/clusters/igad-cluster-1-karamoja-cluster/. Accessed 10 October 2025.

[CR57] Inselman, A.D. 2003. Environmental degradation and conflict in Karamoja, Uganda: The decline of a pastoral society. *International Journal of Global Environmental Issues* 3: 168–187. 10.1504/ijgenvi.2003.003863.

[CR58] IPBES. 2018. The IPBES assessment report on land degradation and restoration. Bonn: IPBES. https://www.ipbes.net/assessment-reports/ldr. Accessed 10 October 2025.

[CR59] IPBES. 2024a. Summary for policymakers of the thematic assessment report on the interlinkages among biodiversity, water, food and health of the intergovernmental science-policy platform on biodiversity and ecosystem services. Bonn: IPBES. 10.5281/zenodo.13850290. Accessed 10 October 2025.

[CR60] IPBES. 2024b. Thematic assessment report on the underlying causes of biodiversity loss and the determinants of transformative change and options for achieving the 2050 vision for biodiversity of the intergovernmental science-policy platform on biodiversity and ecosystem services. Bonn: IPBES. 10.5281/zenodo.11382215. Accessed 10 October 2025.

[CR61] IPC. 2024a. Kenya: IPC acute food insecurity and acute malnutrition analysis. February–June 2024. https://www.ipcinfo.org/ipc-country-analysis/details-map/en/c/1156893/?iso3=KEN. Accessed 10 October 2025.

[CR62] IPC. 2024b. Uganda: IPC acute food insecurity analysis for Karamoja. March 2024–February 2025. https://www.ipcinfo.org/fileadmin/user_upload/ipcinfo/docs/IPC_Uganda_Acute_Food_Insecurity_Malnutrition_Mar_2024_Feb_2025_Report.pdf. Accessed 10 October 2025.

[CR63] IPCC. 2014. Climate change 2014: Impacts, adaptation and vulnerability. In *Part B: Regional aspects. Contribution of working group II to the fifth assessment report of the intergovernmental panel on climate change*. Cambridge, New York: Cambridge University Press. https://www.ipcc.ch/report/ar5/wg2/. Accessed 10 October 2025.

[CR64] IPCC. 2019. Climate change and land: An IPCC special report on climate change, desertification, land degradation, sustainable land management, food security, and greenhouse gas fluxes in terrestrial ecosystems. https://www.ipcc.ch/srccl/. Accessed 10 October 2025.

[CR65] Jain, K., D. Dokka, and T.A. Crane. 2025. Carbon markets in African livestock sectors: A scoping study and research agenda. Nairobi: ILRI. https://cgspace.cgiar.org/items/c90d633a-676c-46d1-b176-362969611e49. Accessed 10 October 2025.

[CR66] Jenet, A., N. Buono, S. Di Lello, M. Gomarasca, C. Heine, S. Mason, M. Nori, R. Saavedra, et al. 2016. The path to greener pastures: Pastoralism, the backbone of the world’s drylands. 10.13140/RG.2.2.11042.22725. Accessed 10 October 2025.

[CR67] Jiren, T.S., D.J. Abson, J. Schultner, M. Riechers, and J. Fischer. 2023. Bridging scenario planning and backcasting: A Q-analysis of divergent stakeholder priorities for future landscapes. *People and Nature* 5: 572–590. 10.1002/pan3.10441.

[CR68] Karamage, F., C. Zhang, T. Liu, A. Maganda, and A. Isabwe. 2017. Soil erosion risk assessment in Uganda. *Forests* 8: 52. 10.3390/f8020052.

[CR69] Karmebäck, V.N., J.N. Wairore, M. Jirström, and G. Nyberg. 2015. Assessing gender roles in a changing landscape: Diversified agro-pastoralism in drylands of West Pokot, Kenya. *Pastoralism* 5: 1–8. 10.1186/s13570-015-0039-4.

[CR70] Kawira, C., A. Hörnell, C. Kunyanga, B. Schumann, M. Okoth, S. Mureithi, and K. Lindvall. 2025. Perspectives on nutritional resilience in a setting with persistent food insecurity in the drylands of West Pokot, Kenya. *Journal of Hunger & Environmental Nutrition*. 10.1080/19320248.2025.2592828.

[CR71] Kilaka, B.M. 2022. *Security controversies along the LAPSSET infrastructure corridor in Kenya*. Gothenburg: University of Gothenburg, Faculty of Social Sciences.

[CR72] Knutsson, P., S. Mureithi, E. Wredle, and G. Nyberg. 2021. Perspectives on enclosures in pastoralist drylands: From contradictory evidence to the formulation of innovative land management strategies. *World Development Perspectives* 23: 100351. 10.1016/j.wdp.2021.100351.

[CR73] Kyanjo, J.L., A. Turinawe, A. Hörnell, and K. Lindvall. 2025. Balancing maternal employment and child nutrition and health: A grounded theory study of rural communities in Northeastern Uganda. *BMC Public Health* 25: 769. 10.1186/s12889-025-21978-z. PMC11853224 40001081

[CR74] Lind, J., R. Sabates-Wheeler, and S. Kohnstamm. 2016. Changes in the drylands of Eastern Africa: A review of evidence and data and their implications for efforts to strengthening resilience. https://opendocs.ids.ac.uk/opendocs/handle/20.500. Accessed 10 October 2025.

[CR75] Lind, J., D. Okenwa, and I. Scoones. 2020a. The politics of land, investments and resources in Eastern Africa’s pastoral drylands. In *Land, investment & politics: Reconfiguring Eastern Africa’s pastoral drylands*, ed. J. Lind, D. Okenwa, and I. Scoones. Suffolk: Boydell & Brewer.

[CR76] Lind, J., R. Sabates-Wheeler, M. Caravani, L.B.D. Kuol, and D.M. Nightingale. 2020b. Newly evolving pastoral and post-pastoral rangelands of Eastern Africa. *Pastoralism* 10: 24. 10.1186/s13570-020-00179-w. PMC7666883 33224462

[CR77] Liniger, H., and W. Critchley. 2007. Where the land is greener: Case-studies and analysis of soil and water conservation initiatives worldwide. CTA/CDE/FAO/UNEP/WOCAT. https://wocat.net/en/wocat-media-library/where-the-land-is-greener/. Accessed 10 October 2025.

[CR78] Liu, J., H. Mooney, V. Hull, S.J. Davis, J. Gaskell, T. Hertel, J. Lubchenco, K.C. Seto, et al. 2015. Systems integration for global sustainability. *Science* 347: 1258832. 10.1126/science.1258832. 25722418

[CR79] Löfqvist, S., F. Kleinschroth, A. Bey, A. de Bremond, R. DeFries, J. Dong, F. Fleischman, S. Lele, et al. 2022. How social considerations improve the equity and effectiveness of ecosystem restoration. *BioScience* 73: 134–148. 10.1093/biosci/biac099. PMC9991587 36896142

[CR80] Maes, S.L., M.P. Perring, R. Cohen, F.K. Akinnifesi, A. Bargués-Tobella, J.-F. Bastin, M. Bauters, P.N. Bernardino, et al. 2024. Explore before you restore: Incorporating complex systems thinking in ecosystem restoration. *Journal of Applied Ecology* 61: 922–939. 10.1111/1365-2664.14614.

[CR81] Maestre, F.T., R. Salguero-Gómez, and J.L. Quero. 2012. It is getting hotter in here: Determining and projecting the impacts of global environmental change on drylands. *Philosophical Transactions of the Royal Society B: Biological Sciences* 367: 3062–3075. 10.1098/rstb.2011.0323. PMC3479685 23045705

[CR82] Maitima, J.M., S.M. Mugatha, R.S. Reid, L.N. Gachimbi, A. Majule, H. Lyaruu, D. Pomery, S. Mathai, et al. 2009. The linkages between land use change, land degradation and biodiversity across East Africa. *African Journal of Environmental Science and Technology* 3: 310–325.

[CR83] Mamdani, M. 1982. Karamoja: Colonial roots of famine in North-East Uganda. *Review of African Political Economy* 9: 66–73. 10.1080/03056248208703517.

[CR84] Masterson, V.A., S. Vetter, T. Chaigneau, T.M. Daw, O. Selomane, M. Hamann, G.Y. Wong, V. Mellegård, et al. 2019. Revisiting the relationships between human well-being and ecosystems in dynamic social-ecological systems: Implications for stewardship and development. *Global Sustainability* 2: e8. 10.1017/sus.2019.5.

[CR85] MEA. 2005. *Ecosystems and human well-being: Synthesis*. Washington: Island Press.

[CR86] Meyerson, S. 2023. “A reason not to belong”: Political decentralization, intercommunal relations, and changing identities in Northeastern Uganda. *African Studies Review* 66: 394–416. 10.1017/asr.2022.128.

[CR87] Mganga, K.Z. 2022. Agricultural land degradation in Kenya. In *Impact of Agriculture on Soil Degradation I: Perspectives from Africa, Asia, America and Oceania*, ed. P. Pereira, M. Munoz-Rojas, I. Bogunovic, and W. Zhao, 273–300. Cham: Springer.

[CR88] Middleton, N., L. Stringer, A. Goudie, and D. Thomas. 2011. The forgotten billion: MDG achievement in the drylands. New York: UNDP-UNCCD. https://www.undp.org/publications/forgotten-billion-mdg-achievement-drylands. Accessed 10 October 2025.

[CR89] Mkutu, K.A. 2008. *Guns and governance in the Rift Valley: Pastoralist conflict and small arms*. London: James Currey.

[CR90] Mokori, A., A.H. Ndungutse, D. Amongin, C.N. Agabiirwe, R. Byabasheija, and J. Draru. 2023. Use of collaborative learning approach for increased cure rate among children aged 6–59 months with severe wasting in Karamoja, Uganda. *BMJ Open Quality* 12: e001941. 10.1136/bmjoq-2022-001941. PMC9923296 36764732

[CR91] Moon, K., D. Marsh, and C. Cvitanovic. 2021. Coupling property rights with responsibilities to improve conservation outcomes across land and seascapes. *Conservation Letters* 14: e12767. 10.1111/conl.12767.

[CR92] Moore, M.-L., and M. Milkoreit. 2020. Imagination and transformations to sustainable and just futures. *Elementa: Science of the Anthropocene*. 10.1525/elementa.2020.081.

[CR93] Moore, M.-L., O. Tjornbo, E. Enfors, C. Knapp, J. Hodbod, J.A. Baggio, A. Norström, P. Olsson, et al. 2014. Studying the complexity of change toward an analytical framework for understanding deliberate social-ecological transformations. *Ecology and Society* 19: 54. 10.5751/ES-06966-190454.

[CR94] Mósesdóttir, L. 2024. Energy (in)justice in the green energy transition: The case of Fosen wind farms in Norway. *Technology in Society* 77: 102563. 10.1016/j.techsoc.2024.102563.

[CR95] Mosley, J., and E.E. Watson. 2016. Frontier transformations: Development visions, spaces and processes in Northern Kenya and Southern Ethiopia. *Journal of Eastern African Studies* 10: 452–475. 10.1080/17531055.2016.1266199.

[CR96] Muhereza, E.F. 2017. Pastoralist and livestock development in Karamoja, Uganda: A rapid review of African regional policy and programming initiatives. Kampala: Karamoja Resilience Support Unit, USAID. https://karamojaresilience.org/wp-content/uploads/2021/05/tufts_1742_krsu_review_policies_karamoja_v3_online.pdf. Accessed 10 October 2025.

[CR97] Mureithi, S., S. Mwendwa, and Y. Ayalew. 2025. Soil and water conservation technologies for ecosystem restoration in drylands. In *Land restoration through ecosystem-based approach: Contexts from drylands of the Global South*, ed. Y.D.I. Khan, S. Purohit, J.M. Jibrin, and M.D. Gholkar, 425–484. Cham: Springer.

[CR98] Mwangi, E. 2007. Subdividing the commons: Distributional conflict in the transition from collective to individual property rights in Kenya’s Maasailand. *World Development* 35: 815–834. 10.1016/j.worlddev.2006.09.012.

[CR99] Myers, N., R.A. Mittermeier, C.G. Mittermeier, G.A.B. da Fonseca, and J. Kent. 2000. Biodiversity hotspots for conservation priorities. *Nature* 403: 853–858. 10.1038/35002501. 10706275

[CR100] Nabweteme, H., G. Bostedt, and A. Turinawe. 2025. Livelihood security shocks and coping strategies in the drylands of Kenya and Uganda—A seasonal analysis. *Development Studies Research* 12: 2516434. 10.1080/21665095.2025.2516434.

[CR101] Nakalembe, C., J. Dempewolf, and C. Justice. 2017. Agricultural land use change in Karamoja Region, Uganda. *Land Use Policy* 62: 2–12. 10.1016/j.landusepol.2016.11.029.

[CR102] Ndiritu, S.W., A.M.G. Waimiri, A. Muthanga, and S. Baskaran-Makanju. 2025. An in-depth assessment of pastoral policy landscape in Kenya. *Pastoralism: Research, Policy and Practice* 15: 14334. 10.3389/past.2025.14334.

[CR103] Ng, W.-T., M. Immitzer, M. Floriansitz, F. Vuolo, L. Luminari, C. Adede, R. Wahome, and C. Atzberger. 2016. Mapping Prosopis spp. within the Tarach water basin, Turkana, Kenya using sentinel-2 imagery. In *Remote sensing for agriculture, ecosystems, and hydrology XVIII*, 161–173. SPIE.

[CR104] Nyaga, M.N., S.M. Mureithi, G. Nyberg, D. Mpairwe, H.L. Gonda, A. Bargués-Tobella, and E. Wredle. 2025. Effects of manuring and grass-legume mixtures on herbaceous vegetation in semi-arid East African rangelands. *African Journal of Range and Forage Science*. 10.2989/10220119.2025.2570704.

[CR105] Nyberg, G., P. Knutsson, M. Ostwald, I. Öborn, E. Wredle, D.J. Otieno, S. Mureithi, P. Mwangi, et al. 2015. Enclosures in West Pokot, Kenya: Transforming land, livestock and livelihoods in drylands. *Pastoralism: Research, Policy and Practice*. 10.1186/s13570-015-0044-7.

[CR106] Odei Obeng-Amoako, G.A., C.A.S. Karamagi, J. Nangendo, J. Okiring, Y. Kiirya, R. Aryeetey, E. Mupere, M. Myatt, et al. 2021. Factors associated with concurrent wasting and stunting among children 6–59 months in Karamoja, Uganda. *Maternal & Child Nutrition* 17: e13074. 10.1111/mcn.13074. PMC7729532 32830434

[CR107] Oduor, F.O., D.W.M. Kaindi, G.O. Abong, F. Thuita, and C. Termote. 2023. Diversity and utilization of indigenous wild edible plants and their contribution to food security in Turkana County, Kenya. *Frontiers in Sustainable Food Systems* 7: 1113771. 10.3389/fsufs.2023.1113771.

[CR108] Oduor, F., G. Keding, D.M. Kaindi, G. Abong, F. Thuita, and C. Termote. 2024. Quantifying Turkana’s wild edible plants’ contribution to critical micronutrient and dietary diversity among women of reproductive age. *CyTA-Journal of Food* 22: 2413949. 10.1080/19476337.2024.2413949.

[CR109] Oduor, F., D.M. Kaindi, G. Abong, F. Thuita, and C. Termote. 2025. Community-based conservation strategies for wild edible plants in Turkana County, Kenya. *Conservation* 5: 1. 10.3390/conservation5010001.

[CR110] Olsson, P., V. Galaz, and W.J. Boonstra. 2014. Sustainability transformations: A resilience perspective. *Ecology and Society* 19: 1. 10.5751/ES-06799-190401.

[CR111] Omondi, S., and M. Odhiambo. 2009. Pastoralism, policies and practice in the Horn and East Africa: A review of current trends. London: Overseas Development Institute. Accessed 10 October 2025.

[CR112] Onyango, P. 2018. Nomadic pastoralism and natural resource use conflicts in East Africa. In *Pastoralism and climate change in East Africa*, ed. P.Z. Yanda and C.G. Mung’ong’o. Dar Es Salaam: Mkuki Na Nyota.

[CR113] Osborne, T., S. Brock, R. Chazdon, S. Chomba, E. Garen, V. Gutierrez, R. Lave, M. Lefevre, et al. 2021. The political ecology playbook for ecosystem restoration: Principles for effective, equitable, and transformative landscapes. *Global Environmental Change* 70: 102320. 10.1016/j.gloenvcha.2021.102320.

[CR114] Pas, A., E.E. Watson, and B. Butt. 2023. Land tenure transformation: The case of community conservancies in northern Kenya. *Political Geography* 106: 102950. 10.1016/j.polgeo.2023.102950.

[CR115] Pasiecznik, N., and C. Reij. 2020. *Restoring African drylands*. Wageningen: Tropenbos International.

[CR116] Pavanello, S. 2009. Pastoralists’ vulnerability in the Horn of Africa: Exploring political marginalization, donors’ policies, and cross-border issues. London: https://www.preventionweb.net/files/24285_24285literaturereviewrread1.pdf. Accessed 10 October 2025.

[CR117] Pereira, L.M., T. Hichert, M. Hamann, R. Preiser, and R. Biggs. 2018. Using futures methods to create transformative spaces: Visions of a good Anthropocene in southern Africa. *Ecology and Society* 23: 19. 10.5751/ES-09907-230119.

[CR118] Powell, J. 2010. Karamoja: A literature review. Safeworld. https://www.files.ethz.ch/isn/121396/2010-03_Karamoja%20A%20literature%20review.pdf. Accessed 10 October 2025.

[CR119] Reid, R.S., M.E. Fernández-Giménez, and K.A. Galvin. 2014. Dynamics and resilience of rangelands and pastoral peoples around the Globe. *Annual Review of Environment and Resources* 39: 217–242. 10.1146/annurev-environ-020713-163329.

[CR120] Republic of Kenya. 2012a. Land (Group Representatives) Act. Laws of Kenya, Chapter 287. Nairobi.

[CR121] Republic of Kenya. 2012b. Land Adjudication Act. Laws of Kenya, Chapter 284. Nairobi.

[CR122] Republic of Kenya. 2016. The Community Land Act. Nairobi.

[CR123] Republic of Uganda. 2000. Republic of Uganda: Plan for modernization of agriculture, Ministry of Agriculture, Animal Industry and Fisheries, Entebbe.

[CR124] Reynolds, J.F., D.M.S. Smith, E.F. Lambin, B.L. Turner, M. Mortimore, S.P.J. Batterbury, T.E. Downing, H. Dowlatabadi, et al. 2007. Global desertification: Building a science for dryland development. *Science* 316: 847–851. 10.1126/science.1131634. 17495163

[CR125] Robbins, P. 2019. *Political ecology: A critical introduction*. Hoboken: Wiley.

[CR126] Robinson, J., S. Burch, S. Talwar, M. O’Shea, and M. Walsh. 2011. Envisioning sustainability: Recent progress in the use of participatory backcasting approaches for sustainability research. *Technological Forecasting and Social Change* 78: 756–768. 10.1016/j.techfore.2010.12.006.

[CR200] Robinson, L., and F. Flintan. 2022. Can formalisation of pastoral land tenure overcome its paradoxes? Reflections from East Africa. *Pastoralism* 12: 34. 10.1186/s13570-022-00250-8.

[CR127] Rugadya, M.A., and H. Kamusiime. 2013. Tenure in mystery: The status of land under wildlife, forestry and mining concessions in Karamoja region, Uganda. *Nomadic Peoples* 17: 33–65. 10.3167/np.2013.170103.

[CR128] Rutten, M.M.E.M. 1992. *Selling wealth to buy poverty: The process of the individualization of landownership among the Maasai pastoralists of Kajiado district, Kenya, 1890–1990*. Breitenbach: Saarbrücken.

[CR129] Safriel, U., Z. Adeel, D. Niemeijer, J. Puigdefabregas, R. White, R. Lal, M. Winslow, J. Ziedler, et al. 2005. Dryland systems. In *Ecosystems and human well-being: Current state and trends—Findings of the condition and trends working group*, ed. R. Hassan, R. Scholes, and N. Ash, 623–662. Washington: Island Press.

[CR130] Schlee, G., and Shongolo, A.A. 2012. *Pastoralism and politics in northern Kenya and southern Ethiopia*. Martlesham: Boydell and Brewer.

[CR131] Schumann, B., T. Alice, L. Kristina, K.J. Lule, K.D. Ansyijar, K. Caroline, M. Annrose, M. Peter, et al. 2025. Livelihood dynamics and challenges to wellbeing in the drylands of rural East Africa—The Drylands transform study population in the Karamoja border region. *Global Health Action* 18: 2490330. 10.1080/16549716.2025.2490330. PMC12024496 40270287

[CR132] Scoones, I., A. Stirling, D. Abrol, J. Atela, L. Charli-Joseph, H. Eakin, A. Ely, P. Olsson, et al. 2020. Transformations to sustainability: Combining structural, systemic and enabling approaches. *Current Opinion in Environmental Sustainability* 42: 65–75. 10.1016/j.cosust.2019.12.004.

[CR133] Scoones, I., M. Nori, S. Bose, R. Gogineni, N. Maru, P. Tsering, G. Simula, T. Mohamed, et al. 2023. *Pastoralism, uncertainty and resilience*. Rugby: Practical Action Publishing.

[CR134] Seneviratne, S.I., X. Zhang, M. Adnan, W. Badi, C. Dereczynski, A. Di Luca, S. Ghosh, I. Iskandar, et al. 2021. Weather and climate extreme events in a changing climate. In *Climate change 2021: The physical science basis. Contribution of working group I to the sixth assessment report of the intergovernmental panel on climate change*, ed. V. Masson-Delmotte, P. Zhai, A. Pirani, S.L. Connors, C. Péan, S. Berger, N. Caud, Y. Chen, et al., 1513–1766. Cambridge, New York: Cambridge University Press.

[CR135] Sietz, D., M.K.B. Lüdeke, and C. Walther. 2011. Categorisation of typical vulnerability patterns in global drylands. *Global Environmental Change* 21: 431–440. 10.1016/j.gloenvcha.2010.11.005.

[CR136] Sigman, E., and M. Elias. 2021. Three approaches to restoration and their implications for social inclusion. *Ecological Restoration* 39: 27–35. 10.3368/er.39.1-2.27.

[CR137] Stein, D., and C. Valters. 2012. *Understanding theory of change in international development*. London: Justice and Security Research Programme, International Development Department, London School of Economics and Political Science.

[CR138] Stojanovic, T., H.M. McNae, P. Tett, T.W. Potts, J. Reis, H.D. Smith, and I. Dillingham. 2016. The “social” aspect of social-ecological systems: A critique of analytical frameworks and findings from a multisite study of coastal sustainability. *Ecology and Society* 21: 15. 10.5751/ES-08633-210315.

[CR139] Swärd, A.L.E. 2023. Ecosystem services from woody vegetation in East African rangelands. MSc thesis. Swedish University of Agricultural Sciences (SLU), Department of Forest Ecology and Management.

[CR140] Szoke-Burke, S., and K.Y. Cordes. 2020. Mechanisms for consultation and free, prior and informed consent in the negotiation of investment contracts. *Northwestern Journal of International Law & Business* 41: 49.

[CR141] Turner, M., and E. Schlecht. 2019. Livestock mobility in sub-Saharan Africa: A critical review. *Pastoralism* 9: 13. 10.1186/s13570-019-0150-z.

[CR142] UN. 2015. Transforming our world: The 2030 agenda for sustainable development. https://sdgs.un.org/publications/transforming-our-world-2030-agenda-sustainable-development-17981. Accessed 10 October 2025.

[CR143] UNDP. 2019. Beyond income, beyond averages, beyond today: Inequalities in human development in the 21st century, New York, USA. https://hdr.undp.org/content/human-development-report-2019. Accessed 10 October 2025.

[CR144] UNEP. 1992. *World atlas of desertification*. London: Edward Arnold.

[CR145] UNESCO & UN-Water. 2020. United Nations world water development report 2020: Water and climate change, Paris. https://www.unwater.org/publications/un-world-water-development-report-2020. Accessed 10 October 2025.

[CR146] UNICEF & WHO. 2019. Progress on household drinking water, sanitation and hygiene 2000–2017: Special focus on inequalities, New York. https://www.unicef.org/reports/progress-on-drinking-water-sanitation-and-hygiene-2019. Accessed 10 October 2025.

[CR147] Upton, C. 2014. The new politics of pastoralism: Identity, justice and global activism. *Geoforum* 54: 207–216. 10.1016/j.geoforum.2013.11.011.

[CR148] Vågen, T.G., and L.A. Winowiecki. 2023. The LDSF field manual-land and soil health assessments using the Land Degradation Surveillance Framework (LDSF). Nairobi: World Agroforestry (ICRAF). https://worldagroforestry.org/output/land-degradation-surveillance-framework-field-manual. Accessed 10 October 2025.

[CR149] Vågen, T.G., and L.A. Winowiecki. 2019. Predicting the spatial distribution and severity of soil erosion in the global tropics using satellite remote sensing. *Remote Sensing* 11: 1800. 10.3390/rs11151800. PMC7771391 33489317

[CR150] Vergragt, P.J., and J. Quist. 2011. Backcasting for sustainability: Introduction to the special issue. *Technological Forecasting and Social Change* 78: 747–755. 10.1016/j.techfore.2011.03.010.

[CR151] Wainaina, P., P.A. Minang, J. Nzyoka, L. Duguma, E. Temu, and L. Manda. 2021. Incentives for landscape restoration: Lessons from Shinyanga, Tanzania. *Journal of Environmental Management* 280: 111831. 10.1016/j.jenvman.2020.111831. 33352378

[CR152] Walker, P. 2017. *Change management for sustainable development*. Lincoln: IEMA.

[CR153] Whitmee, S., A. Haines, C. Beyrer, F. Boltz, A.G. Capon, B.F. de Souza Dias, A. Ezeh, H. Frumkin, et al. 2015. Safeguarding human health in the Anthropocene epoch: Report of The Rockefeller Foundation; Lancet Commission on Planetary Health. *The Lancet* 386: 1973–2028. 10.1016/S0140-6736(15)60901-1. 26188744

[CR154] WHO. 2023. WHO factsheet: Malnutrition. https://www.who.int/news-room/fact-sheets/detail/malnutrition. Accessed 10 October 2025.

[CR155] Williams, B.K. 2011. Adaptive management of natural resources—Framework and issues. *Journal of Environmental Management* 92: 1346–1353. 10.1016/j.jenvman.2010.10.041. 21075505

[CR156] Wily, L.A. 2011. ‘The law is to blame’: The vulnerable status of common property rights in Sub-Saharan Africa. *Development and Change* 42: 733–757. 10.1111/j.1467-7660.2011.01712.x.

[CR157] Winowiecki, L.A., A. Bargués-Tobella, J. Maina, J. Musembi Kimeu, and T.G. Vågen. 2023. Baseline assessment of soil and land health indicators within the ‘Drylands Transform’ and ‘Drylands Restore’ sites in Kenya and Uganda using the LDSF. World Agroforestry (ICRAF). 10.34725/DVN/UJVTK8.

[CR158] Yan, H., H. Yang, X. Guo, S. Zhao, and Q. Jiang. 2022. Payments for ecosystem services as an essential approach to improving ecosystem services: A review. *Ecological Economics* 201: 107591. 10.1016/j.ecolecon.2022.107591.

[CR159] Young, O.R. 2012. Navigating the sustainability transition: Governing complex and dynamic socio-ecological systems. In *Global environmental commons: Analytical and political challenges in building governance mechanisms*, ed. E. Brousseau, T. Dedeurwaerdere, P.-A. Jouvet, and M. Willinger. Oxford: Oxford University Press.

[CR160] Zika, M., and K.-H. Erb. 2009. The global loss of net primary production resulting from human-induced soil degradation in drylands. *Ecological Economics* 69: 310–318. 10.1016/j.ecolecon.2009.06.014.

[CR161] Zomer, R.J., J. Xu, and A. Trabucco. 2022. Version 3 of the global aridity index and potential evapotranspiration database. *Scientific Data* 9: 409. 10.1038/s41597-022-01493-1. PMC9287331 35840601

