# Supplementary material for: Social–ecological transformations towards sustainability in drylands: A conceptual framework with examples from the Karamoja cluster in East Africa
Source: Ambio. 2026 Apr 24;55(11):2663–90. doi: 10.1007/s13280-026-02389-9 (PMC13661849; doi:10.1007/s13280-026-02389-9)
Supplement: Supplementary file 1 [file 13280_2026_2389_MOESM1_ESM.pdf]

Supplementary Information

Title: Social-ecological transformations towards sustainability in drylands – a conceptual framework with examples from the Karamoja cluster in East Africa

Journal: Ambio

Authors: Bargués-Tobella, Aida*; Knutsson, Per; Bostedt, Göran; Hörnell, Agneta; Lindvall, Kristina; Mpairwe, Denis; Mureithi, Stephen; Nyberg, Gert; Nyberg, Ylva; Ong'ao Ng'asike, Philemon ; Schumann, Barbara; Turinawe, Alice; Vågen, Tor Gunnar; Winowiecki, Leigh-Ann; Wredle, Ewa & Öborn, Ingrid

* Corresponding author. Affiliation: Agrotecnio - CERCA Center, Lleida, Spain; Department of Forest Ecology and Management, Swedish University of Agricultural Sciences (SLU), Umeå, Sweden. E-mail: aida.bargues@agrotecnio.udl.cat; aida.bargues.tobella@slu.se

Table S1. Development and wellbeing indicators used in Figure 3, including the raw data, data sources and indicator description (Uganda). All values are expressed as percentages of the relevant population.

**Indicator**	**Description**	**National average**	**National average Urban**	**National average Rural**	**Karamoja sub-region**	**Reference to the data source**
Education: School non-attendance	Proportion of population aged 10 and above that has never attended school	12.9	6.9	14.9	70.8	UBOS. 2017. Education: A means for population transformation. Thematic series. Based on the National Population and Housing Census 2014.
Education: Illiteracy rate	Illiteracy rate (population aged 10 and above)	27.8	14.9	32.2	75.4	UBOS. 2017. Education: A means for population transformation. Thematic series. Based on the National Population and Housing Census 2014.
Energy: Not clean energy sources for cooking	Percentage distribution of conventional HHs not using clean sources of energy for cooking	97.1	NA	NA	98.8	UBOS. 2014. National Population and Housing Census 2014.
Energy: Not connected to electricity grid	Percentage distribution of conventional HHs without connection to a grid	79.8	54.4	92.7	97.9	UBOS. 2022. Uganda National Service Delivery Survey (NSDS) 2021.
Health: Births not in health facility	Percentage of births not in health facility (hospitals, health centres, dispensary/clinic) in the two years preceding the survey	13.6	6.8	16.5	18.2	UBOS. 2023. Uganda Demographic and Health Survey 2022.
Health: Nutritional status children, Moderately stunted	Nutritional status of children under 5. Moderately stunted- Height-for-age, percentage below -2SD	24.4	20.5	25.7	41.1	UBOS. 2023. Uganda Demographic and Health Survey 2022.
Health: Nutritional status children, Severely stunted	Nutritional status of children under 5. Severely stunted - Height-for-age, percentage below -3SD	8.4	7.1	8.8	18	UBOS. 2023. Uganda Demographic and Health Survey 2022.
Health: Nutritional status children, Moderately underweight	Nutritional status of children under 5. Moderately underweight - Weight-for-age, percentage below -2SD	9.7	8.2	10.2	30.4	UBOS. 2023. Uganda Demographic and Health Survey 2022.
Health: Nutritional status children, Severely underweight	Nutritional status of children under 5. Severely underweight - Weight-for-age, percentage below -3SD	2.4	1.7	2.6	11.7	UBOS. 2023. Uganda Demographic and Health Survey 2022.
Health: Nutritional status children, Moderately wasted	Nutritional status of children under 5. Moderately wasted - Weight-for hight, percentage below -2SD	3.2	2.7	3.4	11.9	UBOS. 2023. Uganda Demographic and Health Survey 2022.
Health: Nutritional status children, Severely wasted	Nutritional status of children under 5. Severely wasted - Weight-for-hight, percentage below -3SD	1	0.7	1.1	4.2	UBOS. 2023. Uganda Demographic and Health Survey 2022.
WASH: Unimproved and surface water sources	Percentage distribution of de jure population by drinking water service ladder	18.6	11.7	21.4	8.9	UBOS. 2023. Uganda Demographic and Health Survey 2022.
WASH: No toilet facility (open defecation)	Percentage distribution of households with no toilet facility (open defecation)	5.4	1.7	7.1	61.6	UBOS. 2022. Uganda National Service Delivery Survey (NSDS) 2021.
Poverty: Monetary total	Monetary poverty incidence	21.4	9.6	25	60.2	UBOS. 2018. Uganda National Household Survey 2016/2017

Table S2. Development and wellbeing indicators used in Figure 3, including the raw data, data sources and indicator description (Kenya). All values are expressed as percentages of the relevant population.

Indicator	Description	National average	National average Urban	National average Rural	West Pokot county	Turkana county	Reference to the data source
Education: School non-attendance	Percentage of population above 3 years old that has never attended school	16.3	8.8	19.6	39.6	68.7	KNBS. 2019. Kenya population and housing census. Volume IV: Population by county and sub-county.
Education: Illiteracy rate	Percentage of population aged 15 years and above with no ability to read and write (illiterate)	14.1	5.8	19.5	36.9	59.1	KNBS. 2016. Kenya Integrated Household Budget survey 2015-2016
Energy: Not clean energy sources for cooking	Percentage distribution of conventional HHs not using clean sources of energy for cooking	74.5	44.6	93.4	96.4	96.9	KNBS. 2019. Kenya population and housing census. Volume IV: Population by county and sub-county.
Energy: Not connected to electricity grid	Percentage distribution of conventional HHs without connection to a grid	49.6	11.6	73.7	88.2	91.4	KNBS. 2019. Kenya population and housing census. Volume IV: Population by county and sub-county.
Health: Births not in health facility	Percentage of births not in health facility (hospitals, health centres, dispensary/clinic) in the two years preceding the survey	11.9	3	16.9	40.2	50.5	KNBS. 2023. Kenya Demographic and Health Survey 2022.
Health: Nutritional status children, Moderately stunted	Nutritional status of children under 5. Moderately stunted- Height-for-age, percentage below -2SD	17.6	12.1	20.3	33.5	23	KNBS. 2023. Kenya Demographic and Health Survey 2022.
Health: Nutritional status children, Severely stunted	Nutritional status of children under 5. Severely stunted - Height-for-age, percentage below -3SD	4.2	2.7	4.9	10.4	7.3	KNBS. 2023. Kenya Demographic and Health Survey 2022.
Health: Nutritional status children, Moderately underweight	Nutritional status of children under 5. Moderately underweight - Weight-for-age, percentage below -2SD	10.1	6.7	11.9	27.1	31.7	KNBS. 2023. Kenya Demographic and Health Survey 2022.
Health: Nutritional status children, Severely underweight	Nutritional status of children under 5. Severely underweight - Weight-for-age, percentage below -3SD	1.8	1.2	2.1	6	8.8	KNBS. 2023. Kenya Demographic and Health Survey 2022.
Health: Nutritional status children, Moderately wasted	Nutritional status of children under 5. Moderately wasted - Weight-for hight, percentage below -2SD	4.9	4	5.3	11	22.6	KNBS. 2023. Kenya Demographic and Health Survey 2022.
Health: Nutritional status children, Severely wasted	Nutritional status of children under 5. Severely wasted - Weight-for-hight, percentage below -3SD	0.6	0.7	0.6	0.9	3.5	KNBS. 2023. Kenya Demographic and Health Survey 2022.
WASH: Unimproved and surface water sources	Percentage distribution of de jure population by drinking water service ladder	23.5	6	32.2	57.8	33.9	KNBS. 2023. Kenya Demographic and Health Survey 2022.
WASH: No toilet facility (open defecation)	Percentage distribution of households with no toilet facility (open defecation)	8.4	1.4	13.9	52.5	62.8	KNBS. 2016. Kenya Integrated Household Budget survey 2015-2016
Shocks	Proportion of households that experienced a shock (at least one) within the previous 5 years precedeing the survey	61.9	53.4	68.7	78.9	75.8	KNBS. 2016. Kenya Integrated Household Budget survey 2015-2016
Poverty: Monetary total	Monetary poverty incidence	35.7	29.1	40.1	57.3	78.5	KNBS. 2020. Comprehensive poverty report. Children, youth, women, men & the elderly. From National to County level.
Poverty: Multidimensional total	Multidimensional poverty incidence	53	28	66.9	82.2	86.3	KNBS. 2020. Comprehensive poverty report. Children, youth, women, men & the elderly. From National to County level.
